# Supporting Differentiated Streaming Services in Heterogeneous Vehicle-to-Everything Networks

**DOI:** 10.3390/s24155007

**Published:** 2024-08-02

**Authors:** Chenn-Jung Huang, Kai-Wen Hu, Hao-Wen Cheng, Mei-En Jian, Muhammad Inas Farras Tsamarah

**Affiliations:** 1 Department of Computer Science & Information Engineering, National Dong Hwa University, Shoufeng, Hualien County 974301, Taiwan; 2586611811v@gmail.com (H.-W.C.); jocker2859122@gmail.com (M.-E.J.); inasfarras@gmail.com (M.I.F.T.); 2 Lookout, Inc., Taipei 110207, Taiwan; drive55555@gmail.com

**Keywords:** electric vehicle, V2X, bandwidth allocation, multimedia application, intelligence control

## Abstract

Advancements in assisted driving technologies are expected to enable future passengers to use a wide range of multimedia applications in electric vehicles (EVs). To address the bandwidth demands for high-resolution and immersive videos during peak traffic, this study introduces a bandwidth-management algorithm to support differentiated streaming services in heterogeneous vehicle-to-everything (V2X) networks. By leveraging cellular 6G base stations, along with Cell-Free (CF) Massive Multi-Input Multi-Output (mMIMO) Wi-Fi 7 access points, the algorithm aims to provide a high-coverage, high-speed, and low-interference V2X network environment. Additionally, Li-Fi technology is employed to supply extra bandwidth to vehicles with limited connectivity via V2V communication. Importantly, the study addresses the urgency and prioritization of different applications to ensure the smooth execution of emergency applications and introduces a pre-downloading mechanism specifically for non-real-time applications. Through simulations, the algorithm’s effectiveness in meeting EV users’ bandwidth needs for various multimedia streaming applications is demonstrated. During peak-bandwidth-demand periods, users experienced an average increase in bandwidth of 47%. Furthermore, bandwidth utilization across the V2X landscape is significantly improved.

## 1. Introduction

As assisted driving technology rapidly advances, future electric vehicles (EVs) will transcend their role as mere transportation devices, transforming into mobile offices and entertainment hubs. EVs will support various multimedia applications, enhancing in-car experiences. Notably, 360° video technology will offer immersive viewing, allowing users to freely rotate their perspective and experience panoramic video. This innovation promises a new level of engagement for multimedia applications. Additionally, 360° video calls for remote surgeries represent a groundbreaking telemedicine application [1], offering timely medical assistance and potentially saving lives.

Although multimedia applications provide EV passengers with rich entertainment and information, encoding technology is essential for efficient bandwidth and storage use. High-Efficiency Video Coding (HEVC) is widely used in fields like video surveillance and healthcare for its high-quality compression and fast processing [2], while Versatile Video Coding (VVC) offers significant bitrate reductions compared to HEVC and meets the demands of emerging media [3]. However, VVC’s higher complexity makes it unsuitable for real-time encoding [4]. Despite its longer encoding times, VVC achieves a compression ratio 50% higher than HEVC while maintaining the same video quality [5].

The rise of Vehicle-to-Everything (V2X) communication marks a new era in vehicular interactions, enabling collaboration between vehicles and with infrastructure [6]. As demand for high-resolution and 360° video grows, the V2X network must meet higher performance standards. The upcoming 6G network is set to provide the needed improvements, offering faster, more reliable connections, greater bandwidth, and lower latency [7]. Operating across a wide range of frequencies, including millimeter waves (mmWave), sub-6GHz, terahertz (THz), and visible light [8], 6G will ensure a smooth and efficient multimedia experience in V2X network environments.

Light Fidelity (Li-Fi) technology, which uses visible light for wireless communication, has gained significant attention for its ultra-high-speed data-transmission capabilities. By rapidly modulating LEDs, Li-Fi can achieve fast audio and video transmission [9]. Future EVs could use Li-Fi through headlights for vehicle-to-vehicle (V2V) communication [10]. In the literature, Saranya et al. [11] utilized embedded sensors and control units, employing Li-Fi technology to address challenges in the vehicular environment. They claimed that Li-Fi is most suitable for vehicle wireless communication compared to other wireless communication methods. Yahia et al. [12] improved visible light V2V systems by integrating biconvex and plano-concave lenses to enhance imaging receiver performance.

Beyond the visible light spectrum, the terahertz band stands out as a key frequency range for 6G. It provides ample spectrum resources capable of supporting extremely high data transfer rates [13], making it particularly well-suited for V2X applications. Recent studies have addressed the challenge of supporting V2X with THz technology. Li et al. [14] studied joint sensing and communication in THz V2X and vehicle connectivity issues. They proposed a dynamic graph neural network (GNN) model that selects suitable graph information aggregation functions based on the V2X network topology. This model enhances the extraction of V2X information and increases the capacity of THz services to serve more vehicles. Chang et al. [15] introduced a joint communication and control algorithm for beam alignment in mmWave/THz V2X networks. Their work focused on analyzing the beam alignment process for transmissions from the base station to the vehicle. Rashee and Hu [16] presented an innovative V2X routing approach that leverages Cognitive Radio and Software Defined Networking (SDN) to achieve ultra-high data rates. This method employs predictive routing to intelligently switch between mmWave and THz frequencies.

However, the communication range of THz is relatively short [17], necessitating collaboration with other frequency bands to address its limitations. Wi-Fi 7, recently acclaimed, is a promising option. Operating in unlicensed frequency bands, Wi-Fi technology provides various cost-effective user devices and access points [18]. Wi-Fi 7 will support 320 MHz bandwidth channels and utilize 4096 Quadrature Amplitude Modulation (QAM) to enhance transmission rates [19]. Additionally, Wi-Fi 7 will feature multi-link operation, which improves throughput and transmission reliability and reduces latency by unifying and coordinating link management [20].

With the development of wireless communication technologies, Cell-Free (CF) Massive Multi-Input Multi-Output (mMIMO) technology has emerged to address the limitations of traditional cellular networks. In CF mMIMO systems, all access points are connected to an access point control unit via fronthaul links. These control units, which can be interconnected directly or through the core network, analyze system performance, provide real-time feedback, and manage baseband signals, supporting user-centric transmission [21]. A subset of access points can collectively serve a group of devices on the same time-frequency resources, enhancing signal power and reducing interference [22]. This architecture facilitates seamless communication channel switching and is highly suitable for V2X networks and hotspot coverage [23]. Scholars have advocated for the implementation of CF mMIMO in V2X contexts. Zhang et al. [24] introduced a beam alignment technique employing broad learning-assisted CF mMIMO at both user and access-point terminals, conducting simulations to assess user mobility in V2X environments. Kurma et al. [25] thoroughly investigated the robustness of CF mMIMO for V2X networks. Their findings highlight the significant impact of various system parameters on performance, including the transmit power, vehicle mobility, channel state information accuracy, fronthaul link quality, and more.

Numerous scholars have recently proposed research on multimedia application transmission in V2X networks. Dai et al. [26] proposed an algorithm that addresses video quality selection, resource allocation, and vehicle grouping management. They employed multicast and Scalable Video Coding for real-time video transmission in V2X networks. Chowdhury et al. [27] proposed a distributed algorithm to enhance traffic offloading from base stations and reduce the control packet overhead. Their work aims to improve the service efficiency and user satisfaction of real-time video streaming services in V2X networks. Ahmed et al. [28] proposed an intelligent real-time multimedia traffic shaping system for 5G V2X networks using distributed reinforcement learning. This system optimizes coding parameters, including the quantization parameters, group of pictures, and frame rate, to effectively control and shape multimedia traffic. Go and Shin [29] presented a raw-format real-time video streaming framework aimed at reducing latency in V2X networks. Their framework dynamically adjusts the number of Real-time Transport Protocol (RTP) packets and the frame rate to optimize the streaming process. Benzerogue et al. [30] proposed the multi-path transmission protocol for video streaming using a fog computing architecture. This protocol effectively manages network resources to enhance video streaming performance and reliability in V2X environments. Chowdhury et al. [31] introduced an affordable cooperative video streaming solution tailored for infotainment services over heterogeneous V2X networks. They developed a novel multicast protocol optimized for dynamic scenarios to improve streaming data distribution.

From the reviewed literature, it is clear that many recent studies have introduced various frameworks and solutions to optimize the transmission performance of multimedia applications in V2X networks. However, these studies predominantly rely on 5G technology, overlooking the substantial advantages that emerging 6G technologies, Wi-Fi 7, and CF mMIMO offer for enhancing V2X networks. They also fail to explore the potential of wireless communication technologies for V2V multimedia application transmission. Furthermore, the current literature often focuses on a single application type and fails to comprehensively address bandwidth prioritization for different types of applications. As multimedia applications used by vehicle passengers rapidly increase, managing potential bandwidth shortages during peak traffic periods becomes crucial. To ensure that emergency applications receive adequate bandwidth priority, it is essential to design bandwidth allocation schemes that account for the distinct characteristics and requirements of various multimedia applications.

The main contributions of this research are outlined below:

- This study utilizes advanced technologies of wireless communication, including mmWave, sub-6GHz, and THz waves, to deliver higher transmission rates. It also deploys CF mMIMO architecture Wi-Fi 7 access points in congested areas to boost communication reliability, enhance transmission rates, and minimize interference. Through the integration of these advanced network technologies, this study aims to create a robust V2X network environment for EV users.
- This study categorizes multimedia applications based on their characteristics and assigns bandwidth allocation priority accordingly. Bandwidth is sourced from base stations and CF mMIMO access points to prioritize emergency applications lacking sufficient resources. Once emergency applications are adequately addressed, bandwidth is then allocated to real-time applications to ensure they receive satisfactory bandwidth. Additionally, Li-Fi is utilized for V2V communication, allowing EVs with limited bandwidth to access bandwidth from base stations and access points on less-congested road segments via inter-vehicle communication. Lastly, if bandwidth remains insufficient, the study dynamically adjusts video frame rate and resolution based on the type of multimedia application to reduce bandwidth requirements.
- This study proposes a pre-download strategy for non-real-time applications at less congested road segments. EVs download additional video segments anticipated for future playback when the base station or CF mMIMO access point at the passing road segments has sufficient bandwidth. The algorithm dynamically adjusts the bandwidth allocated to vehicle users based on the number of upcoming video segments to be played on the EV. This ensures that each user of non-real-time applications can pre-store a sufficient number of video segments. By leveraging the remaining bandwidth of the base stations and the CF mMIMO access points at less congested road segments, this approach achieves improved overall bandwidth efficiency.

## 2. Optimizing Bandwidth Allocation for Multimedia Applications in V2X Networks

**Figure 1:**
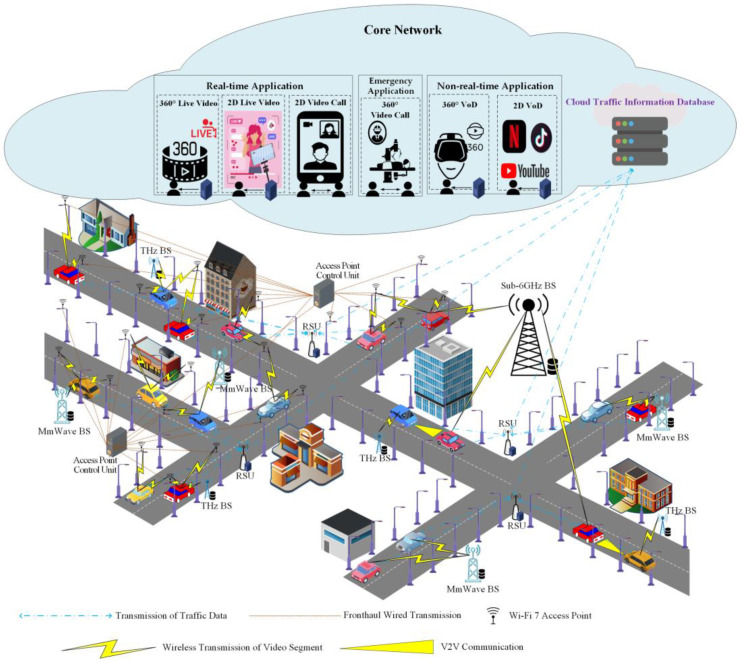
Enhancing differentiated streaming services in diverse V2X networks: a scenario.

To reduce the computational complexity of traditional centralized control architectures, a decentralized computational framework is adopted, as shown in Figure 1. Base stations operating across THz, millimeter-wave, and sub-6GHz frequency bands are strategically positioned along all road segments to serve as bandwidth sources for multimedia applications. To address the high deployment costs associated with densely placing access points and antennas in a CF mMIMO architecture, Wi-Fi 7 access points featuring CF mMIMO technology are selectively deployed solely on streetlights along congested road segments. Each congested road segment is equipped with a CF mMIMO access point control unit, to which all access points on the corresponding road segment are connected. These control units utilize real-time data collected from roadside units (RSUs) on the respective road segments to manage control and coordination. This approach enhances the effective selection of suitable access points for EV users on the same road segment, forming subsets of access points to ensure seamless connectivity, broader signal coverage, and efficient resource allocation.

This study categorizes the multimedia applications used by EV passengers into six types: 2D video on demand (VoD), 2D video calls, 2D live video, 360° VoD, 360° video calls, and 360° live video. These applications are further divided into three categories based on their application levels: emergency application, real-time applications, and non-real-time applications.

- Emergency applications, such as 360° video calls, are essential in scenarios like remote surgery in an ambulance. The broader range of visible angles provided by 360° video calls is crucial for performing precise operations, ensuring that medical professionals can view and assess the situation comprehensively.
- Real-time applications include 2D video calls, 2D live video, and 360° live video
- Non-real-time applications include 2D VoD and 360° VoD.

Notably, both emergency and real-time applications utilize High Efficiency Video Coding (HEVC) due to its lower encoding complexity [2], while non-real-time applications employ Versatile Video Coding (VVC) for its higher compression rates [5].

In our algorithm, EV users plan their routes with assistance from an onboard system installed in the EV prior to departure. Once the user sets the destination, time, and departure location, the “Real-Time Traffic and Travel Time Calculation” module is activated. This module calculates the most suitable route based on the EV user’s preferences and uploads the travel time for each segment to the RSUs along the route.

When an EV user starts a multimedia application, the roadside units (RSUs) along the EV’s route are notified of the multimedia application requirements and vehicle information. The RSUs then contact their respective CF mMIMO access point control units to coordinate the access points, allocating bandwidth to each application accordingly. If the EV moves outside the coverage area of access points in the CF mMIMO architecture or the allocated bandwidth by the access points is insufficient, the managing RSUs request bandwidth for the vehicular multimedia application from the base stations along its route instead. In scenarios where the multimedia applications of EV users cannot meet their bandwidth requirements while driving through specific congested road segments, the EV requests the RSUs managing those segments to locate other EVs that can form a fleet to forward the bandwidth from CF mMIMO access points or base stations from less congested road segments. Notably, Li-Fi technology is utilized for V2V communication within the fleet to assist in forwarding the required bandwidth for multimedia applications.

If EV users initiate multimedia applications while the EV is in motion, the “Multimedia Application Bandwidth Adjustment” module is activated. This module interfaces with the multimedia application software provider’s server to obtain the relevant specifications and requirements. The multimedia application quality settings provided by the server, along with user requirements set by EV users, are then uploaded to the RSUs along the route. Based on the type of multimedia application, the RSUs activate either the “Emergency/Real-time Application Bandwidth Assignment” module or the “Non-Real-Time Application Download” module.

When an EV is within the communication range of access points in the CF mMIMO architecture, the application quality settings and user requirements are communicated to the CF mMIMO access point control units through the RSUs. The control units select and form clusters of access points to serve the EV users, prioritizing bandwidth for emergency/real-time applications. If certain clusters of access points cannot meet the bandwidth requirements of the multimedia applications during rush hours, or if the EV is not within the communication range of access points in the CF mMIMO architecture, the RSUs will request the base station(s) in the covered road segment to support the bandwidth demand of the multimedia applications.

Moreover, if bandwidth is still available, the non-real-time applications can pre-fetch additional video segments by utilizing the remaining bandwidth. This pre-fetching process ensures a smooth multimedia experience for users, even when the EV is not within the coverage range of CF mMIMO architectures and base stations later along the route. The bandwidth allocated from base stations and CF mMIMO access points is dynamically adjusted based on the remaining video segments stored on the EV.

In cases where the base stations and CF mMIMO clusters along the managed route segment cannot provide sufficient bandwidth for EV passengers, the “Emergency/Real-time Application Bandwidth Assignment” module prioritizes emergency and real-time applications by reallocating bandwidth initially assigned to VoD applications. If the bandwidth-demanding applications still face a deficit, the managing RSU activates the “V2V Bandwidth Forwarding” module. This module facilitates V2V communication to forward available bandwidth from base stations or the CF mMIMO architecture located in uncongested segments. Initially, the managing RSU in the bandwidth-deficient road segment will form an EV fleet based on its real-time traffic database and assist in harvesting bandwidth from base stations or CF mMIMO access points in less congested segments.

This work periodically tracks the quality of multimedia application usage for EV passengers. During peak hours, if there is a need to downgrade the quality of these applications, the “Multimedia Application Bandwidth Adjustment” module will be activated. This module adjusts the frame rate of multimedia applications to reduce bandwidth requirements and determines whether to change the resolution based on the application’s characteristics. Emergency applications, due to the critical nature of their functions, such as maintaining accuracy in remote surgery, will not be subjected to resolution reduction. They will always be prioritized in bandwidth reallocation to ensure sufficient bandwidth availability. If bandwidth is insufficient, the bandwidth initially allocated to real-time and non-real-time applications will be reassigned to emergency applications. Specifically, real-time and non-real-time applications will have their resolution adjusted in a timely manner to reduce bandwidth requirements, thereby avoiding interruptions in playback due to bandwidth reduction.

The detailed descriptions of each module are as follows:

### 2.1. Real-Time Traffic and Travel Time Calculation

This module is activated before the EV commences its journey. Once the EV passenger enters the destination information, time, and departure location into the onboard system, the EV initiates its journey. Initially, this module utilizes historical road traffic information from the EV database. Leveraging the potential of machine learning techniques to accurately predict driving times on roads [32,33], this module adopts support vector regression (SVR) technology [32] to estimate the travel time for each road segment based on the historical data of the EV. This process generates routes that align with the driving preferences of the EV user.

Subsequently, this module obtains real-time traffic information for each road segment of the route from the corresponding RSUs to determine the EV’s arrival times at all road segments along the route. Taking into account that the EV passengers might change their itinerary at the last minute or that factors like yielding to emergency vehicles might affect the arrival times and driving speeds compared to previously estimated times, this module recalculates the arrival times for each segment based on the latest traffic conditions upon arriving at the next intersection along its route. When there are significant deviations in the arrival times of the road segments compared to the initial estimates, this module reports the updated arrival times to the corresponding RSUs. The traffic condition information kept at each RSU is then adjusted accordingly.

**Figure 2:**
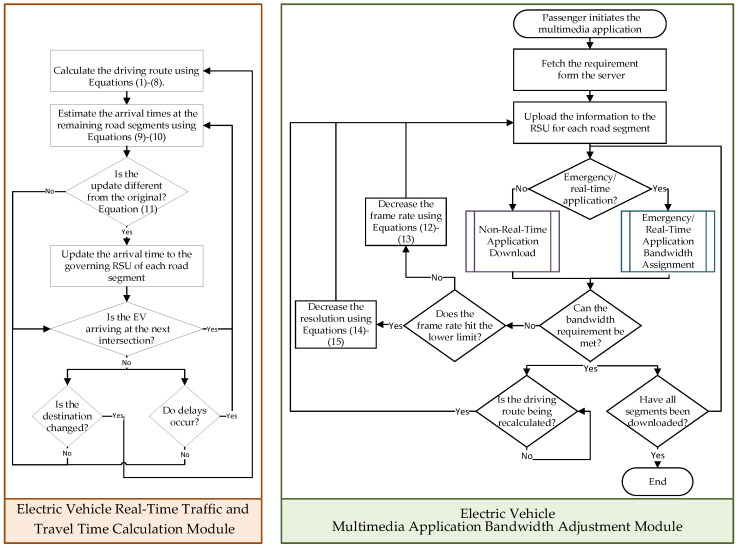
Flow charts of “Real-time Traffic and Travel Time Calculation” module and “Multimedia Application Bandwidth Adjustment” module.

The flowchart of this module is illustrated on the left side of Figure 2, and the execution steps are outlined below:

Step 1: After setting the departure time, location, and destination, this module calculates the most suitable route for the EV user’s preferences as follows:

(1) $$\underset{l}{\arg}\;\mathrm{Min}\left[\omega_{1}^{\sigma }\cdot \sum_{1\leq i\leq \varphi^{\sigma }}\sum_{1\leq j<h_{l,i}^{\sigma }}{sl}_{c_{l,i,j}^{\sigma },c_{l,i,j+1}^{\sigma }}+\omega_{2}^{\sigma }\cdot \sum_{1\leq i\leq \varphi^{\sigma }}\sum_{1\leq j<h_{l,i}^{\sigma }}{cp}_{c_{l,i,j}^{\sigma },c_{l,i,j+1}^{\sigma }}\left({rt}_{c_{l,i,j}^{\sigma }}\right)\;+\omega_{3}^{\sigma }\cdot \left(rt_{c_{l,\varphi^{\sigma },h_{l,\varphi^{\sigma }}^{\sigma }}^{\sigma }}-{rt}_{c_{l,1,1}^{\sigma }}\right)\right],$$

subject to the following:

(2) $$R_{l}^{\sigma }=\left\{R_{l,1}^{\sigma },R_{l,2}^{\sigma },\cdots ,R_{l,i}^{\sigma },R_{l,i+1}^{\sigma },\cdots ,R_{l,\varphi^{\sigma }}^{\sigma }\right\},\;1\leq i\leq \varphi^{\sigma }$$

(3) $$R_{l,i}^{\sigma }=\left(c_{l,i,1}^{\sigma },c_{l,i,2}^{\sigma },\cdots ,c_{l,i,h_{l,i}^{\sigma }-1}^{\sigma },c_{l,i,h_{l,i}^{\sigma }}^{\sigma }\right),1\leq i\leq \varphi^{\sigma }$$

(4) $$c_{l,1,1}^{\sigma }=Org,\;c_{l,\varphi^{\sigma },h_{l,\varphi^{\sigma }}^{\sigma }}^{\sigma }=Dst,$$

(5) $$rt_{c_{l,1,1}^{\sigma }}={rt}_{Org}^{\sigma },$$

(6) $${sl}_{c_{l,i,j}^{\sigma },c_{l,i,j+1}^{\sigma }}=\infty ,\;if\;\xi_{c_{l,i,j}^{\sigma },c_{l,i,j+1}^{\sigma }}\left({rt}_{c_{i}^{l}}\right)=1,\;1\leq i\leq \varphi^{\sigma },1\leq j<h_{l,i}^{\sigma },$$

(7) $${rt}_{c_{l,i,j+1}^{\sigma }}={rt}_{c_{l,i,j}^{\sigma }}+{SD}_{c_{l,i,j}^{\sigma },c_{l,i,j+1}^{\sigma }}\left({rt}_{c_{l,i,j}^{\sigma }}\right)+{ID}_{c_{l,i,j}^{\sigma },c_{l,i,j+1}^{\sigma }}\left({rt}_{c_{l,i,j}^{\sigma }}\right),\;1\leq i\leq \varphi^{\sigma },1\leq j<h_{l,i}^{\sigma },$$

(8) $${rt}_{c_{l,i+1,1}^{\sigma }}={rt}_{c_{l,i,h_{l,i}^{\sigma }}^{\sigma }}+{PD}_{c_{l,i,h_{l,i}^{\sigma }}^{\sigma },c_{l,i+1,1}^{\sigma }}\left({rt}_{c_{l,i,h_{l,i}^{\sigma }}^{\sigma }}\right)+{ID}_{c_{l,i,h_{l,i}^{\sigma }}^{\sigma },c_{l,i+1,1}^{\sigma }}\left({rt}_{c_{l,i,h_{l,i}^{\sigma }}^{\sigma }}\right),\;1\leq i<\varphi^{\sigma },$$

The parameters are as follows:

(i) Equation (1) selects the optimal route tailored to the preferences of the EV passenger. The Min (·) operation takes three parameters in sequence: the total distance traveled by the EV from the departure location to the destination, the congestion pricing for controlled road segments during peak hours, and the travel time required to reach the destination, including any intermediate waypoints that EV σ must pass through. EV passengers can adjust the weighting values of $\omega_{1}^{\sigma }$, $\omega_{2}^{\sigma }$, and $\omega_{3}^{\sigma }$ based on their preset preferences.
(ii) This module constructs the *l*-th candidate route $R_{l}^{\sigma }$ for the EV according to the intermediate stopping points planned by the EV user, such as purchasing meals, household items, or picking up colleagues along the route, etc. $R_{l,i}^{\sigma }$ represents the driving route from the (*i* − 1)-th stopping point to the *i*-th stopping point for σ, where $\varphi^{\sigma }$ is the number of stopping points along the route from the departure location to the destination for σ.
(iii) *Org* and *Dst* represent the positions of the starting point and destination, respectively. For the candidate route indexed by *l*, $c_{l,1,1}^{\sigma }$ denotes the starting point, $c_{l,i,h_{l,i}^{\sigma }}^{\sigma }$ indicates the *i*-th intermediate stopping point, and $c_{l,\varphi^{\sigma },h_{l,\varphi^{\sigma }}^{\sigma }}^{\sigma }$ represents the final destination. ${sl}_{c_{l,i,j}^{\sigma },c_{l,i,j+1}^{\sigma }}$ denotes the length of the connecting road segment between two intersections, $c_{l,i,j}^{\sigma }$ and $c_{l,i,j+1}^{\sigma }$. ${rt}_{c_{l,i,j}^{\sigma }}$ is the time when the EV arrives at intersection $c_{l,i,j}^{\sigma }$, while ${rt}_{Org}^{\sigma }$ and ${rt}_{Dst}^{\sigma ,max}$, respectively, represent the departure time of the EV and the latest acceptable time for the EV user to arrive at the destination.
(iv) ${cp}_{c_{l,i,j}^{\sigma },c_{l,i,j+1}^{\sigma }}\left({rt}_{c_{l,i,j}^{\sigma }}\right)$ represents the congestion pricing implemented for the road segment between intersections $c_{l,i,j}^{\sigma }$ and $c_{l,i,j+1}^{\sigma }$ at the time ${rt}_{c_{l,i,j}^{\sigma }}$ when the EV arrives. If the EV does not pass through any toll road segments or if it arrives at $c_{l,i,j}^{\sigma }$ during off-peak hours, the value of ${cp}_{c_{l,i,j}^{\sigma },c_{l,i,j+1}^{\sigma }}\left({rt}_{c_{l,i,j}^{\sigma }}\right)$ is set to zero. ${SD}_{c_{l,i,j}^{\sigma },c_{l,i,j+1}^{\sigma }}\left({rt}_{c_{l,i,j}^{\sigma }}\right)$ denotes the time spent by the EV to pass through the road segment connecting $c_{l,i,j}^{\sigma }$ and $c_{l,i,j+1}^{\sigma }$ after arriving at $c_{l,i,j}^{\sigma }$ at time ${rt}_{c_{l,i,j}^{\sigma }}$. ${ID}_{c_{l,i,j}^{\sigma },c_{l,i,j+1}^{\sigma }}\left({rt}_{c_{l,i,j}^{\sigma }}\right)$ represents the time required for the EV to enter the road segment connecting $c_{l,i,j}^{\sigma }$ and $c_{l,i,j+1}^{\sigma }$ after arriving at $c_{l,i,j}^{\sigma }$ at time ${rt}_{c_{l,i,j}^{\sigma }}$ due to possible traffic control, and ${PD}_{c_{l,i,h_{l,i}^{\sigma }}^{\sigma },c_{l,i+1,1}^{\sigma }}\left({rt}_{c_{l,i,h_{l,i}^{\sigma }}^{\sigma }}\right)$ is the time spent by the EV at the intermediate stopping point $c_{l,i,h_{l,i}^{\sigma }}^{\sigma }$. Here, the ${SD}_{c_{l,i,j}^{\sigma },c_{l,i,j+1}^{\sigma }}\left(\cdot \right)$, ${ID}_{c_{i}^{l},c_{i+1}^{l}}$, and ${PD}_{c_{l,i,h_{l,i}^{\sigma }}^{\sigma },c_{l,i+1,1}^{\sigma }}\left(\cdot \right)$ are all predicted using SVR technology.
(v) The binary flag $\xi_{c_{l,i,j}^{\sigma },c_{l,i,j+1}^{\sigma }}\left({rt}_{c_{l,i,j}^{\sigma }}\right)$ is used to control whether the road segment connecting $c_{l,i,j}^{\sigma }$ and $c_{l,i,j+1}^{\sigma }$ allows the EV to pass at time ${rt}_{c_{l,i,j}^{\sigma }}$. For example, during peak hours, if the traffic control center designates the road segment between $c_{l,i,j}^{\sigma }$ and $c_{l,i,j+1}^{\sigma }$ as a key controlled segment and prohibits the EV from driving on it, then $\xi_{c_{l,i,j}^{\sigma },c_{l,i,j+1}^{\sigma }}\left({rt}_{c_{l,i,j}^{\sigma }}\right)$ is set to 1; otherwise, it is set to 0.

Step 2: Upon arriving at the next intersection along its route, EV σ requests the latest traffic information for the remaining road segments from the corresponding RSUs. It then updates its estimated arrival times at the remaining intersections on the route accordingly:

(9) $${\hat{rt}}_{c_{l,i,j+1}^{\sigma }}={\hat{rt}}_{c_{l,i,j}^{\sigma }}+{SD}_{c_{l,i,j}^{\sigma },c_{l,i,j+1}^{\sigma }}\left({\hat{rt}}_{c_{l,i,j}^{\sigma }}\right)+{ID}_{c_{l,i,j}^{\sigma },c_{l,i,j+1}^{\sigma }}\left({\hat{rt}}_{c_{l,i,j}^{\sigma }}\right),\;1\leq i\leq \varphi^{\sigma },1\leq j<h_{l,i}^{\sigma },$$

(10) $${\hat{rt}}_{c_{l,i+1,1}^{\sigma }}={\hat{rt}}_{c_{l,i,h_{l,i}^{\sigma }}^{\sigma }}+{PD}_{c_{l,i,h_{l,i}^{\sigma }}^{\sigma },c_{l,i+1,1}^{\sigma }}\left({\hat{rt}}_{c_{l,i,h_{l,i}^{\sigma }}^{\sigma }}\right)+{ID}_{c_{l,i,h_{l,i}^{\sigma }}^{\sigma },c_{l,i+1,1}^{\sigma }}\left({\hat{rt}}_{c_{l,i,h_{l,i}^{\sigma }}^{\sigma }}\right),\;1\leq i<\varphi^{\sigma },$$

where ${\hat{rt}}_{c_{l,i,j}^{\sigma }}$ is the updated arrival time at intersection $c_{l,i,j}^{\sigma }$. ${SD}_{c_{l,i,j}^{\sigma },c_{l,i,j+1}^{\sigma }}\left({\hat{rt}}_{c_{l,i,j}^{\sigma }}\right)$ denotes the time that the EV traverses the road segment connecting $c_{l,i,j}^{\sigma }$ and $c_{l,i,j+1}^{\sigma }$ after arriving at $c_{l,i,j}^{\sigma }$ at time ${\hat{rt}}_{c_{l,i,j}^{\sigma }}$, and ${ID}_{c_{l,i,j}^{\sigma },c_{l,i,j+1}^{\sigma }}\left({\hat{rt}}_{c_{l,i,j}^{\sigma }}\right)$ denotes the time spent at the intersection $c_{l,i,j}^{\sigma }$ due to traffic control after arriving at time ${\hat{rt}}_{c_{l,i,j}^{\sigma }}$. ${PD}_{c_{l,i,h_{l,i}^{\sigma }}^{\sigma },c_{l,i+1,1}^{\sigma }}\left({\hat{rt}}_{c_{l,i,h_{l,i}^{\sigma }}^{\sigma }}\right)$ represents the time spent by the EV at intermediate stopping point $c_{l,i,h_{l,i}^{\sigma }}^{\sigma }$.

Step 3: Compare the calculated arrival times of the EV at each road segment to the originally expected times and check if they exceed the system’s predefined threshold value ϵ:

(11) $${dat}_{c_{l,i,j}^{\sigma }}^{\sigma }=\left\{\begin{array}{cc} 1 & if\;\left|{\hat{rt}}_{c_{l,i,j}^{\sigma }}-{rt}_{c_{l,i,j}^{\sigma }}\right|\geq \epsilon \cdot ∆\\ 0 & others \end{array}\right.,{cp}^{\sigma }\leq i\leq \varphi^{\sigma },1\leq j<h_{l,i}^{\sigma }$$

where ${rt}_{c_{l,i,j}^{\sigma }}$ and ${\hat{rt}}_{c_{l,i,j}^{\sigma }}$ represent the originally predicted arrival time and the updated estimated arrival time for EV σ at intersection $c_{l,i,j}^{\sigma }$, respectively. ∆ is the time slot interval, ${cp}^{\sigma }$ denotes the road segment currently being traversed by the EV, and ϵ is a constant predefined by the system.

Step 4: If ${dat}_{p_{j}^{\sigma }}^{\sigma }=1$, then notify the RSU responsible for the road segment of the adjusted arrival time.

Step 5: Switch to background execution mode.

Step 6: If the destination has not yet been reached, the module will go back to Step 2 and continue execution before arriving at each road segment along the route.

Step 7: If the EV encounters emergency vehicles, such as ambulances or police cars that need to be yielded to, or if delays occur due to traffic congestion along the driving route, this module will return to Step 4 to recalculate the arrival times for each remaining road segment before proceeding to the next intersection.

Step 8: If the EV passenger makes a last-minute change to the itinerary, this module will return to Step 1 to recalculate the most appropriate driving route starting from the current location.

### 2.2. Multimedia Application Bandwidth Adjustment

When an EV passenger initiates a multimedia application while the EV is in motion, this module retrieves the application’s specifications from the multimedia-application-provider’s server. It then uploads these specifications to the RSU along the driving route and notifies the corresponding RSU to activate the bandwidth-allocation module according to the type of application used by the EV passenger. Additionally, this module addresses bandwidth supply–demand imbalances during peak hours by dynamically reducing the frame rate or decreasing the video resolution of applications. However, to ensure that critical applications, such as remote surgeries, are not affected by resolution reduction that could lead to operation faults, this module will maintain the resolution of emergency applications unchanged. This approach prioritizes the accuracy and reliability of emergency applications while ensuring the equitable allocation of bandwidth resources to address the different requirements of various applications, particularly under resource constraints.

The flowchart of this module is illustrated on the right side of Figure 2, and the execution steps are outlined below:

Step 1: After the multimedia application is launched, this module retrieves the relevant specifications and requirements of the application from the server of the multimedia application provider.

Step 2: This module uploads the driving times of the EV on each road segment, as well as the requirements and specifications of the multimedia application, to the RSU of the corresponding road segment.

Step 3: If the multimedia application type used by the EV passenger is for emergency or real-time purposes, the “Emergency/Real-time Application Bandwidth Assignment” module is activated. Conversely, the “Non-Real-Time Application Download” module is activated.

Step 4: If the multimedia application’s bandwidth requirements can be met, advance to Step 8 of the process.

Step 5: If the frame rate of the multimedia application used by the EV passenger has not decreased to the preset minimum, then reduce the frame rate as follows to decrease its bandwidth requirements:

(12) $${uf}_{t_{j}^{\sigma ,v}}=\left\{\begin{array}{cc} {vf}_{f^{\sigma ,v}-1} & if\;{ursu}_{t_{j}^{\sigma ,v}}>0,\;f^{\sigma ,v}>1,\;{at}_{p_{i}^{\sigma }}\leq t_{j}^{\sigma ,v}\leq {at}_{p_{i+1}^{\sigma }},\;1\leq j\leq e^{\sigma ,v},1\leq i<h^{\sigma }\\ {\underline{f}}^{v} & others \end{array}\right.$$

(13) $${df}_{t_{j}^{\sigma ,v}}=\left\{\begin{array}{cc} {vf}_{f^{\sigma ,v}-1} & if\;{drsu}_{t_{j}^{\sigma ,v}}>0,\;f^{\sigma ,v}>1,\;{at}_{p_{i}^{\sigma }}\leq t_{j}^{\sigma ,v}\leq {at}_{p_{i+1}^{\sigma }},\;1\leq j\leq e^{\sigma ,v},1\leq i<h^{\sigma }\\ {\underline{f}}^{v} & others \end{array}\right.$$

where ${ursu}_{t_{j}^{\sigma ,v}}$ and $d{drsu}_{t_{j}^{\sigma ,v}}$ designate upload and download bandwidth insufficiencies, respectively, for EV σ traverses at time slot $t_{j}^{\sigma ,v}$. $t_{1}^{\sigma ,v}$ and $t_{e^{\sigma ,v}}^{\sigma ,v}$ represent the times when application *v* starts and ends. Additionally, $p_{i}^{\sigma }$ represents the *i*-th junction along the route, and $p_{h^{\sigma }}^{\sigma }$ stands for the endpoint of the route. ${\underline{f}}^{v}$ denotes the minimum frame rate of the application preset by the system for the EV passenger, and ${vf}_{f^{\sigma ,v}}$ represents the streaming frame rate requirement set by the EV passenger at level $f^{\sigma ,v}$.

Step 6: If the application used by the EV passenger is for real-time/non-real-time purposes and the resolution is not equal to the preset minimum value, then reduce the resolution of the real-time/non-real-time application as follows to decrease its bandwidth requirements:

(14) $${uvr}_{t_{j}^{\sigma ,v}}=\left\{\begin{array}{cc} {vr}_{q^{\sigma ,v}-1} & if\;{ursu}_{t_{j}^{\sigma ,v}}>0,\;q^{\sigma ,v}>1,\;{at}_{p_{i}^{\sigma }}\leq t_{j}^{\sigma ,v}\leq {at}_{p_{i+1}^{\sigma }},\;1\leq j\leq e^{\sigma ,v},1\leq i<h^{\sigma }\\ {\underline{r}}^{v} & others \end{array}\right.$$

(15) $${dvr}_{t_{j}^{\sigma ,v}}=\left\{\begin{array}{cc} {vr}_{q^{\sigma ,v}-1} & if\;{drsu}_{t_{j}^{\sigma ,v}}>0,\;q^{\sigma ,v}>1,\;{at}_{p_{i}^{\sigma }}\leq t_{j}^{\sigma ,v}\leq {at}_{p_{i+1}^{\sigma }},\;1\leq j\leq e^{\sigma ,v},1\leq i<h^{\sigma }\\ {\underline{r}}^{v} & others \end{array}\right.$$

where ${\underline{r}}^{v}$ represents the minimum resolution of the application set by the system for the EV passenger, while ${vr}_{q^{\sigma ,v}}$ stands for the expected resolution requirement of the screen at level $q^{\sigma ,v}$ set by the EV passenger.

Step 7: If adjustments to the frame rate or resolution of the application have been made in the preceding steps, return to Step 2 to resume the bandwidth allocation process. Otherwise, inform the EV passenger that the bandwidth requirements cannot be met.

Step 8: This module operates in the background.

If the multimedia application used by the EV passenger is for emergency or real-time purposes, this step verifies whether the EV passenger changes the itinerary temporarily or if there is a significant deviation between the arrival times at the passing-by road segments and the originally estimated times. In such cases, the “Real-Time Traffic Conditions and Travel Time Calculation” module is activated to recalculate the driving route or the times of arrival at segments along the route. The process then proceeds back to Step 2 to resume.

As for VoD applications, if the pre-downloading is complete, this module will stop execution. Otherwise, the RSU responsible for the next road segment is notified, and Step 3 is executed.

### 2.3. Emergency/Real-Time Application Bandwidth Assignment

This module first attempts to allocate bandwidth for emergency and real-time applications based on whether the EV is within the coverage area of a CF mMIMO architecture. If the EV is within this coverage area, the RSU will notify the corresponding CF mMIMO access point control unit. This control unit will then use a clustering algorithm, as described in [34], to group appropriate access points into a cluster that prioritizes bandwidth allocation for the emergency or real-time applications. Notably, if the CF mMIMO access point control unit cannot provide sufficient bandwidth, the RSU will request additional bandwidth from the base station(s) in the covered road segment.

If the EV is not within the coverage area of any CF mMIMO architecture, the RSU will check whether the base stations along the route can provide the necessary bandwidth for the emergency or real-time application. If none of the base stations within the EV’s communication range can provide sufficient bandwidth, the RSU will examine whether these base stations can reallocate bandwidth initially allocated to non-real-time applications. If bandwidth reallocation is performed but the requirements for emergency or real-time applications remain unsatisfied, the RSU will activate the “V2V Bandwidth Forwarding” module to obtain bandwidth from base stations or CF mMIMO architectures in less congested areas. Given that emergency applications should have the highest priority for bandwidth allocation, if the V2V bandwidth forwarding still cannot meet the bandwidth demands for emergency applications, the bandwidth initially allocated to real-time applications will also be reallocated to the emergency applications.

**Figure 3:**
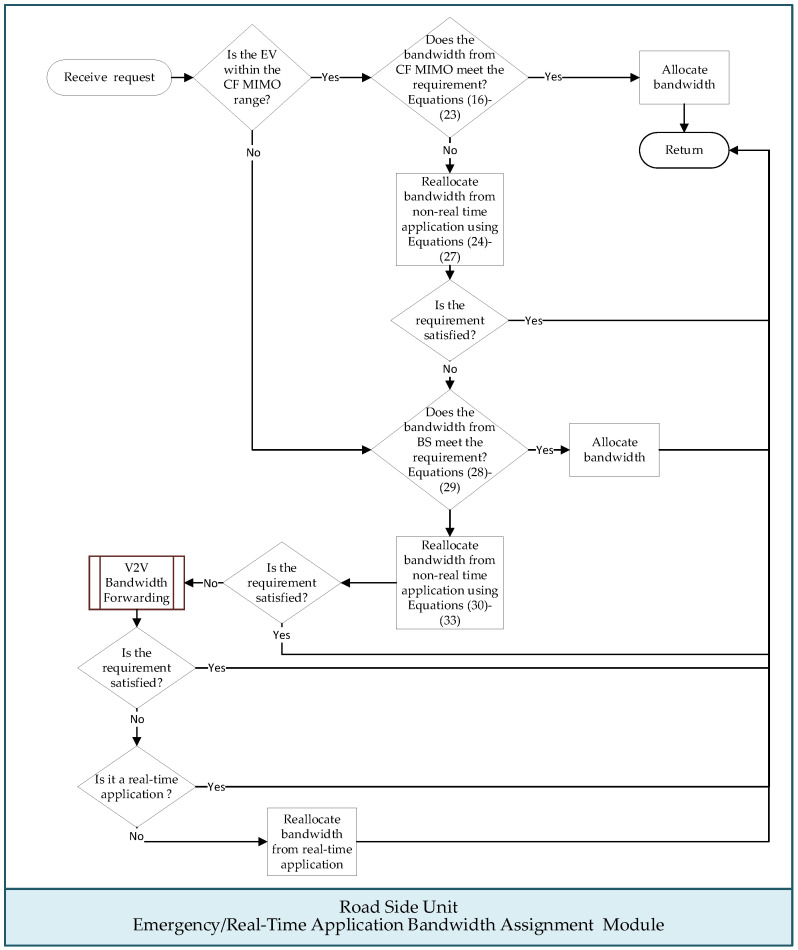
Flow chart of “Emergency/Real-Time Application Bandwidth Assignment” module.

Figure 3 illustrates the flowchart of this module, and the execution steps are outlined below:

Step 1: If the EV is not within the coverage area of any CF mMIMO architecture, advance to Step 9.

Step 2: Upon receiving the bandwidth allocation request, the managing CF mMIMO access point control unit employs an access point clustering algorithm from the literature [34] to select appropriate access points. These selected access points form a cluster to serve the EV passenger.

Step 3: Considering the time that the EV spends on the passing road segments and the specifications of an emergency or real-time application set by EV passengers, the required bandwidth for the application during each time slot is calculated. Specifically, the total bandwidth available from the access point cluster serving the application is examined to determine if it meets the minimum bandwidth requirements for emergency or real-time applications as follows:

(16) $${dub}_{t_{j}^{\sigma ,v}}=\gamma^{\sigma ,v}\cdot \left[\sum_{\vartheta^{\sigma ,v}}{ub}_{t_{j}^{\sigma ,v}}^{\vartheta^{\sigma ,v}}-{uf}_{t_{j}^{\sigma ,v}}\cdot {ur}_{t_{j}^{\sigma ,v}}\right],\;1\leq j\leq e^{\sigma ,v}$$

(17) $${ursu}_{t_{j}^{\sigma ,v}}=\left\{\begin{array}{cc} {rsu}_{p_{i}^{\sigma }} & if\;{dub}_{t_{j}^{\sigma ,v}}<0,\;{at}_{p_{i}^{\sigma }}\leq t_{j}^{\sigma ,v}\leq {at}_{p_{i+1}^{\sigma }},\;1\leq j\leq e^{\sigma ,v},1\leq i<h^{\sigma }\\ 0 & others \end{array}\right.$$

(18) $${ddb}_{t_{j}^{\sigma ,v}}=\sum_{\vartheta^{\sigma ,v}}{db}_{t_{j}^{\sigma ,v}}^{\vartheta^{\sigma ,v}}-{df}_{t_{j}^{\sigma ,v}}\cdot {dr}_{t_{j}^{\sigma ,v}},\;1\leq j\leq e^{\sigma ,v}$$

(19) $${drsu}_{t_{j}^{\sigma ,v}}=\left\{\begin{array}{cc} {rsu}_{p_{i}^{\sigma }} & {if\;{ddb}_{t_{j}^{\sigma ,v}}<0,\;at}_{p_{i}^{\sigma }}\leq t_{j}^{\sigma ,v}\leq {at}_{p_{i+1}^{\sigma }},\;1\leq j\leq e^{\sigma ,v},1\leq i<h^{\sigma }\\ 0 & others \end{array}\right.$$

(20) $${ur}_{t_{j}^{\sigma ,v}}={dr}_{t_{j}^{\sigma ,v}}={vr}_{q^{\sigma ,v}},1\leq j<e^{\sigma ,v},1\leq q^{\sigma ,v}\leq {\bar{q}}^{v}$$

(21) $${uf}_{t_{j}^{\sigma ,v}}={df}_{t_{j}^{\sigma ,v}}={vf}_{f^{\sigma ,v}},1\leq j<e^{\sigma ,v},1\leq f^{\sigma ,v}\leq {\bar{f}}^{v}$$

(22) $$t_{j+1}^{\sigma ,v}=t_{j}^{\sigma ,v}+∆,1\leq j<e^{\sigma ,v}$$

(23) $${at}_{p_{1}^{\sigma }}\leq t_{1}^{\sigma ,v}{\leq t}_{e^{\sigma ,v}}^{\sigma ,v}\leq {at}_{p_{h^{\sigma }}^{\sigma }}$$

where the binary indicator $\gamma^{\sigma ,v}$ represents whether the emergency/real-time application v is a bidirectional application. A value of $\gamma^{\sigma ,v}=1$ indicates that the application is bidirectional, while a value of $\gamma^{\sigma ,v}=0$ indicates that the application is unidirectional. The start and ending time of *v* are respectively denoted by $t_{1}^{\sigma ,v}$ and $t_{e^{\sigma ,v}}^{\sigma ,v}$. $p_{i}^{\sigma }$ indicates the *i*-th intersection index along the driving route, while $p_{h^{\sigma }}^{\sigma }$ represents the destination. ${ub}_{t_{j}^{\sigma ,v}}^{\vartheta^{\sigma ,v}}$ and ${db}_{t_{j}^{\sigma ,v}}^{\vartheta^{\sigma ,v}}$ are used to denote bandwidth for uploading and downloading that $\vartheta^{\sigma ,v}$, a member of the access point cluster, can allocate to the bidirectional application *v* when EV σ passes through the transmission range of $\vartheta^{\sigma ,v}$ during time slot $t_{j}^{\sigma ,v}$. ${ur}_{t_{j}^{\sigma ,v}}$ and ${dr}_{t_{j}^{\sigma ,v}}$ represent the upload and download resolution of *v*, respectively, while ${uf}_{t_{j}^{\sigma ,v}}$ and ${df}_{t_{j}^{\sigma ,v}}$ denote the frame rate of *v* for upload and download, respectively. The RSU responsible for the road segment $p_{i}^{\sigma }$ traversed by the EV is denoted by ${rsu}_{p_{i}^{\sigma }}$. When the values of ${dub}_{t_{j}^{\sigma ,v}}$ and ${ddb}_{t_{j}^{\sigma ,v}}$ are both less than zero, it means that the access point cluster is unable to meet the minimum upload or download bandwidth requirements for application *v* as *σ* traverses the road segment. ${ursu}_{t_{j}^{\sigma ,v}}$ and $d{drsu}_{t_{j}^{\sigma ,v}}$, respectively, represent the RSU managing the road segment $p_{i}^{\sigma }$ where the EV experiences insufficient bandwidth uploading and downloading during time slot $t_{j}^{\sigma ,v}$. The application streaming resolution can be categorized into ${\bar{q}}^{v}$ levels, while ${vr}_{q^{\sigma ,v}}$ denotes the EV passenger’s expected streaming resolution for level $q^{\sigma ,v}$. Similarly, the frame rate of the application can be categorized into ${\bar{f}}^{v}$ levels, while ${vf}_{f^{\sigma ,v}}$ denotes the frame rate expected by the EV passenger for level $f^{\sigma ,v}$.

Step 4: If the access point cluster can satisfy the minimum bandwidth requirements of the emergency or real-time application, the access point control unit should be notified to allocate the bandwidth to the application, and this module will conclude. Otherwise, advance to the subsequent step.

Step 5: The access point control unit reallocates the bandwidth to the demanding emergency or real-time application from that initially assigned for other non-real-time applications:

(24) $${bub}_{t_{j}^{\sigma ,v}}=\left\{\begin{array}{cc} \sum_{\mu }\sum_{\theta^{\mu }}{ub}_{t_{j}^{\sigma ,v}}^{\theta^{\mu }} & \;if\;{dub}_{t_{j}^{\sigma ,v}}<0\\ 0 & others \end{array}\right.,\;1\leq j\leq e^{\sigma ,v}$$

(25) $${dub}_{t_{j}^{\sigma ,v}}={dub}_{t_{j}^{\sigma ,v}}+{bub}_{t_{j}^{\sigma ,v}}$$

(26) $${bdb}_{t_{j}^{\sigma ,v}}=\left\{\begin{array}{cc} \sum_{\mu }\sum_{\theta^{\mu }}{db}_{t_{j}^{\sigma ,v}}^{\theta^{\mu }} & \;if\;{ddb}_{t_{j}^{\sigma ,v}}<0,\\ 0 & others \end{array}\;\right.,\;1\leq j\leq e^{\sigma ,v}$$

(27) $${ddb}_{t_{j}^{\sigma ,v}}={ddb}_{t_{j}^{\sigma ,v}}+{bdb}_{t_{j}^{\sigma ,v}}$$

where the designated non-real-time application is indexed by μ, and $\theta^{\mu }$ denotes a member of the access point control unit responsible for allocating bandwidth to μ. The bandwidth that can be reallocated from non-real-time applications are denoted as ${bub}_{t_{j}^{\sigma ,v}}$ for upload and ${bdb}_{t_{j}^{\sigma ,v}}$ for download.

Step 6: If either of ${bub}_{t_{j}^{\sigma ,v}}$ or ${bdb}_{t_{j}^{\sigma ,v}}$ obtained from the previous step is not equal to zero, it implies that the bandwidth originally allocated by the member $\theta^{\mu }$ of the access point control unit to non-real-time application μ is reassigned to the demanding emergency or real-time application. Accordingly, this step invokes the “Non-Real-Time Application Download” module to assist in downloading the expected video segments for the non-real-time application if either of ${bub}_{t_{j}^{\sigma ,v}}$ or ${bdb}_{t_{j}^{\sigma ,v}}$ is not zero.

Step 7: Check whether the emergency or real-time application meets the minimum bandwidth requirements. If satisfied, the result is sent back to the EV and the module will conclude. Otherwise, advance to the subsequent step.

Step 8: If either of ${dub}_{t_{j}^{\sigma ,v}}$ or ${ddb}_{t_{j}^{\sigma ,v}}$ obtained from Equations (25) and (27) is less than zero, the bandwidth-demanding application still has a deficit. The following equations evaluate whether the base station’s signal coverage area can meet the bandwidth requirements for emergency or real-time applications:

(28) $${dub}_{t_{j}^{\sigma ,v}}={dub}_{t_{j}^{\sigma ,v}}+\sum_{\varphi^{\sigma ,v}}{ub}_{t_{j}^{\sigma ,v}}^{\varphi^{\sigma ,v}},\;if\;{dub}_{t_{j}^{\sigma ,v}}<0,$$

(29) $${ddb}_{t_{j}^{\sigma ,v}}={ddb}_{t_{j}^{\sigma ,v}}+\sum_{\varphi^{\sigma ,v}}{db}_{t_{j}^{\sigma ,v}}^{\varphi^{\sigma ,v}},\;if\;{ddb}_{t_{j}^{\sigma ,v}}<0,$$

where $\varphi^{\sigma ,v}$ is the index of the base station that provides bandwidth to the application.

Step 9: Check whether the emergency or real-time application meets the minimum bandwidth requirements. If satisfied, the result is sent back to the EV and the module will conclude. Otherwise, advance to the subsequent step.

Step 10: Request the base stations that have assigned bandwidth to non-real-time applications to reallocate it to the emergency or real-time application:

(30) $${bub}_{t_{j}^{\sigma ,v}}=\left\{\begin{array}{cc} \sum_{\mu }\sum_{\varphi^{\mu }}{ub}_{t_{j}^{\sigma ,v}}^{\varphi^{\mu }} & \;if\;{dub}_{t_{j}^{\sigma ,v}}<0\\ 0 & others \end{array}\right.,\;1\leq j\leq e^{\sigma ,v}$$

(31) $${dub}_{t_{j}^{\sigma ,v}}={dub}_{t_{j}^{\sigma ,v}}+{bub}_{t_{j}^{\sigma ,v}}$$

(32) $${bdb}_{t_{j}^{\sigma ,v}}=\left\{\begin{array}{cc} \sum_{\mu }\sum_{\varphi^{\mu }}{db}_{t_{j}^{\sigma ,v}}^{\varphi^{\mu }} & \;if\;{ddb}_{t_{j}^{\sigma ,v}}<0,\\ 0 & others \end{array}\right.,\;1\leq j\leq e^{\sigma ,v}$$

(33) $${ddb}_{t_{j}^{\sigma ,v}}={ddb}_{t_{j}^{\sigma ,v}}+{bdb}_{t_{j}^{\sigma ,v}}$$

where the designated non-real-time application is indexed by μ, and $\varphi^{\mu }$ is the base station responsible for allocating bandwidth to μ.

Step 11: If either ${bub}_{t_{j}^{\sigma ,v}}$ or ${bdb}_{t_{j}^{\sigma ,v}}$ is not equal to zero, this step invokes the “Non-Real-Time Application Download” module to assist in harvesting the bandwidth that was initially assigned to non-real-time applications.

Step 12: Upon satisfying the bandwidth demands, the result is returned to the EV, and this module will conclude. Otherwise, advance to the subsequent step.

Step 13: The RSU activates the “V2V Bandwidth Forwarding” module to search for base stations or CF mMIMO access points that can provide bandwidth and relay the bandwidth to the demanding emergency/real-time application via V2V communication.

Step 14: The result is returned to the EV if the bandwidth requirement is met, and then, this module will conclude. Otherwise, advance to the subsequent step.

Step 15: If the multimedia application used by the EV passenger is an emergency application, move on to the next step. Otherwise, the result is returned to the EV, and then, this module will conclude.

Step 16: Reassign the bandwidth that was originally assigned to other real-time applications by all base stations within the signal coverage area of the EV during the same time period to the emergency applications.

Step 17: If any bandwidth that was originally assigned to other real-time applications is reassigned, activate the “V2V Bandwidth Forwarding” module to harvest bandwidth for the real-time applications.

Step 18: Return the result to the EV, and then, this module will conclude.

### 2.4. Non-Real-Time Application Download

Leveraging the ability to pre-generate and store segments of non-real-time applications on the supplier’s server, an EV can preload upcoming video segments into its onboard storage from base stations or access point clusters along the road segments it travels. This preloading can occur while non-real-time application video segments are being played. If the EV is within the coverage area of any access points in a CF mMIMO architecture, the managing CF mMIMO access point control unit will use a clustering algorithm, as detailed in [34], to select appropriate access points for forming a cluster to preload segments for the non-real-time application. However, if the access point cluster cannot provide sufficient bandwidth or if the EV is out of range of the CF mMIMO architecture, the managing RSU will request additional bandwidth support from the base stations serving that road segment. If the allocated bandwidth is still inadequate to preload segments before their scheduled playtime, the “V2V Bandwidth Forwarding” module will be activated to obtain additional bandwidth from CF mMIMO access points or base stations located in less congested road segments.

**Figure 4:**
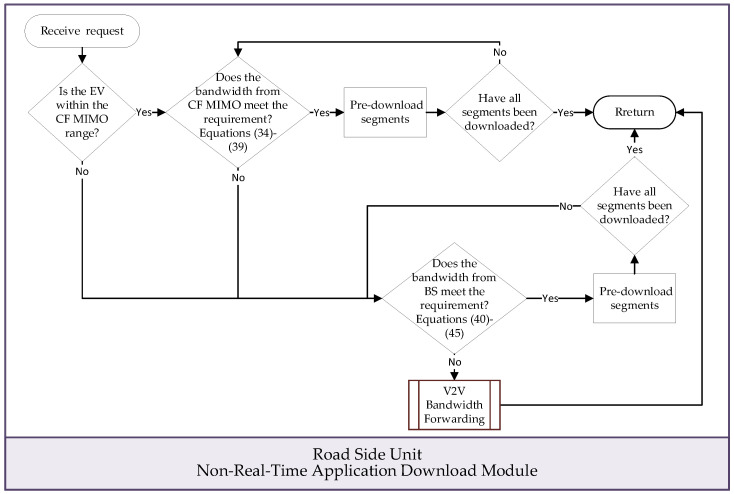
Flow chart of “Non-Real-Time Application Download” module.

Figure 4 illustrates the flowchart of this module, and the execution steps are outlined below:

Step 1: If the EV is not within the coverage area of a CF mMIMO architecture, advance to Step 6 of the process.

Step 2: The CF mMIMO control unit utilizes an access point clustering algorithm, as described in literature [34], to identify suitable access points for forming a cluster.

Step 3: The CF mMIMO cluster preloads application segments according to the specifications of the non-real-time application as follows:

(34) $$\sum_{\delta }\left(∆\cdot \sum_{\vartheta^{\sigma }}{db}_{\tau_{c^{\sigma ,v}}+\delta \cdot ∆}^{\vartheta^{\sigma ,v}}\right){\geq CS}_{c^{\sigma ,v}}\left({{cfr}_{c^{\sigma ,v}},cbr}_{c^{\sigma ,v}},{cd}_{c^{\sigma ,v}}\right),\;1\leq c^{\sigma ,v}\leq C^{\sigma ,v}$$

(35) $$c{pt}_{1}^{,v}\leq \tau_{c^{,v}}<\tau_{c^{\sigma ,v}}+\delta \cdot ∆<c{pt}_{c^{\sigma ,v}}^{\sigma ,v},\;1\leq c^{\sigma ,v}\leq C^{\sigma ,v}$$

(36) $${cpt}_{c^{\sigma ,v}-1}^{\sigma ,v}\leq {cpt}_{c^{\sigma ,v}}^{\sigma ,v},\;1\leq c^{\sigma ,v}\leq C^{\sigma ,v}$$

(37) $${cbr}_{c^{\sigma ,v}}={br}_{q^{\sigma ,v}},\;1\leq c^{\sigma ,v}\leq C^{\sigma ,v}$$

(38) $${rt}_{p_{1}^{\sigma }}\leq {cpt}_{1}^{\sigma ,v}$$

(39) $${buf}_{\tau_{c^{\sigma ,v}}}^{\sigma }+{CS}_{c^{\sigma ,v}}\left({{cfr}_{c^{\sigma ,v}},cbr}_{c^{\sigma ,v}},{cd}_{c^{\sigma ,v}}\right)\leq {\bar{buf}}^{\sigma },$$

$c^{\sigma ,v}$ denotes the downloading segment and $\tau_{c^{\sigma ,v}}$ denotes the time, ${db}_{\tau_{c^{\sigma ,v}}}^{\vartheta^{\sigma ,v}}$ represents the bandwidth allocated to application segment $c^{\sigma ,v}$ during time $\tau_{c^{\sigma ,v}}$ over Δ time slots, and $\vartheta^{\sigma ,v}$ is a member of the CF mMIMO access point. δ signifies the number of consecutive time slots for segment downloads after time $\tau_{c^{\sigma ,v}}$. $C^{\sigma ,v}$ is the total number of segments in the non-real-time application. The playback time of $c^{\sigma ,v}$ is denoted by ${cpt}_{c^{\sigma ,v}}^{\sigma ,v}$, while ${CS}_{c^{\sigma ,v}}\left(\cdot \right)$ stands for the bit rate of $c^{\sigma ,v}$. Additionally, ${cfr}_{c^{\sigma ,v}}$, ${cbr}_{c^{\sigma ,v}}$, and ${cd}_{c^{\sigma ,v}}$ represent the frame rate, resolution, and playback duration of video segment $c^{\sigma ,v}$, respectively. The start and stop times of the non-real-time application are denoted by ${cpt}_{1}^{\sigma ,v}$ and ${cpt}_{c^{\sigma ,v}}^{\sigma ,v}$, respectively, and ${br}_{q^{\sigma ,v}}$ is the resolution requirement set by the EV user for video frame $q^{\sigma ,v}$. The EV will start playing the non-real-time application after arriving at intersection $p_{1}^{\sigma }$, and ${rt}_{p_{1}^{\sigma }}$ is the time when the EV arrives at intersection $p_{1}^{\sigma }$. ${buf}_{\tau_{c^{\sigma ,v}}}^{\sigma }$ denotes σ’s remaining buffer at time $\tau_{c^{\sigma ,v}}$, and ${\bar{buf}}^{\sigma }$ represents the size of σ’s buffer.

Step 4: Once all segments for the non-real-time application being played by the EV passenger have been successfully downloaded, the EV will be notified of the completion, and then, the module will conclude. Otherwise, advance to the subsequent step.

Step 5: The execution returns to step 3 to resume operation if the CF mMIMO cluster is capable of satisfying the minimum bandwidth requirements for pre-downloading non-real-time application content to the EV.

Step 6: All base stations within the EV’s communication range are instructed to allocate bandwidth to pre-download application segments to the fullest extent possible:

(40) $$\sum_{\delta }\left(∆\cdot \sum_{\varphi^{\sigma ,v}}{db}_{\tau_{c^{\sigma ,v}}+\delta \cdot ∆}^{\varphi^{\sigma ,v}}\right){\geq CS}_{c^{\sigma ,v}}\left({{cfr}_{c^{\sigma ,v}},cbr}_{c^{\sigma ,v}},{cd}_{c^{\sigma ,v}}\right),\;1\leq c^{\sigma ,v}\leq C^{\sigma ,v}$$

(41) $$c{pt}_{1}^{\sigma ,v}\leq \tau_{c^{\sigma ,v}}<\tau_{c^{\sigma ,v}}+\delta \cdot ∆<c{pt}_{c^{\sigma ,v}}^{\sigma ,v},\;1\leq c^{\sigma ,v}\leq C^{\sigma ,v}$$

(42) $${cpt}_{c^{\sigma ,v}-1}^{\sigma ,v}\leq {cpt}_{c^{\sigma ,v}}^{\sigma ,v},\;1\leq c^{\sigma ,v}\leq C^{\sigma ,v}$$

(43) $${cbr}_{c^{\sigma ,v}}={br}_{q^{\sigma ,v}},\;1\leq c^{\sigma ,v}\leq C^{\sigma ,v},$$

(44) $${rt}_{p_{1}^{\sigma }}\leq {cpt}_{1}^{\sigma ,v}$$

(45) $${buf}_{\tau_{c^{\sigma ,v}}}^{\sigma }+{CS}_{c^{\sigma ,v}}\left({{cfr}_{c^{\sigma ,v}},cbr}_{c^{\sigma ,v}},{cd}_{c^{\sigma ,v}}\right)\leq {\bar{buf}}^{\sigma },$$

where ${db}_{\tau_{c^{\sigma ,v}}}^{\varphi^{\sigma ,v}}$ represents the bandwidth allocated by base station $\varphi^{\sigma ,v}$ to non-real-time application segment $c^{\sigma ,v}$ during the time period $\tau_{c^{\sigma ,v}}$ over Δ.

Step 7: Once all segments of the non-real-time application being played by the EV passenger have been successfully downloaded, the EV will be informed of the completion, and the module will conclude.

Step 8: If the EV encounters difficulties in smoothly downloading non-real-time application segments due to inadequate bandwidth within its signal coverage area from the base stations, the “V2V Bandwidth Forwarding” module is activated. This module identifies base stations and CF mMIMO clusters situated in less congested road segments to acquire the required bandwidth for preloading. Afterwards, the bandwidth is relayed through V2V communication.

Step 9: The outcome is communicated to the EV passenger. Following this, the module completes its execution.

### 2.5. V2V Bandwidth Forwarding Module

When the RSU receives notification from an EV indicating that the required bandwidth for multimedia applications cannot be met, it initiates a connection between the requesting EV and other EVs within the V2V communication range. The last EV in the fleet can obtain the bandwidth from CF mMIMO clusters or base stations over less congested road segments. Subsequently, the collected bandwidth is transmitted from the last EV in the fleet to the requesting EV via Li-Fi for V2V communication. This cooperative approach ensures that the requesting EV can effectively support multimedia applications for its passengers and efficiently utilize the bandwidth of base stations and CF mMIMO clusters on uncongested road segments.

**Figure 5:**
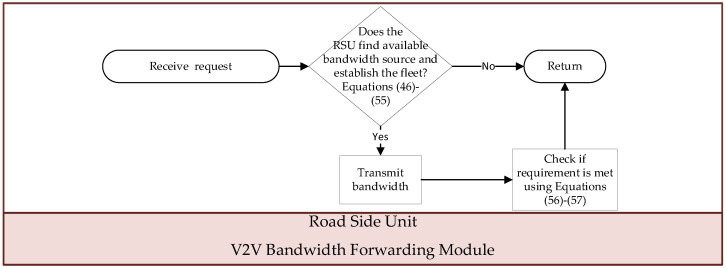
Flow chart of “V2V Bandwidth Forwarding” module.

Figure 5 illustrates the flowchart of this module, and the execution steps are outlined below:

Step 1: To support the bandwidth requirements of the EV passenger’s application, the managing RSU first queries other RSUs on uncongested road segments to identify base stations and CF mMIMO clusters that can offer bandwidth. It then creates connections between vehicles in the vehicle chain.

(46) $$\underset{S^{\sigma ,v}}{arg}Min\left\{\left|\alpha_{t_{j}^{\sigma ,v}}\cdot \left(\sum_{s=1}^{S^{\sigma ,v}}\sum_{\phi^{r_{s,1}^{\sigma ,v}}}{ub}_{t_{l}^{\sigma ,v}}^{\phi^{r_{s,1}^{\sigma ,v}}}-\sum_{\phi^{\sigma }}{ub}_{t_{l}^{\sigma ,v}}^{\phi^{\sigma }}\right)\right|+\beta_{t_{j}^{\sigma ,v}}\;\cdot \left|\sum_{s=1}^{S^{\sigma ,v}}\sum_{\phi^{r_{s,1}^{\sigma ,v}}}{db}_{t_{l}^{\sigma ,v}}^{\phi^{r_{s,1}^{\sigma ,v}}}-\sum_{\phi^{\sigma }}{db}_{t_{l}^{\sigma ,v}}^{\phi^{\sigma }}\right|\right\},$$

subject to the following:

(47) $$R_{s}^{\sigma ,v}=\left(r_{s,1}^{\sigma ,v},r_{s,2}^{\sigma ,v},\cdots ,r_{s,\eta_{s}^{\sigma ,v}-1}^{\sigma ,v},r_{s,\eta_{s}^{\sigma ,v}}^{\sigma ,v}\right),\;1\leq s<S^{\sigma ,v},$$

(48) $$r_{s,\eta_{s}^{\sigma ,v}}^{\sigma ,v}=\sigma ,\;1\leq s<S^{\sigma ,v},$$

(49) $$\alpha_{t_{j}^{\sigma ,v}}=\left\{\begin{array}{cc} 1 & \;{if\;\sigma^{\sigma ,v}=1\;\&\;rt}_{p_{k}^{\sigma }}\leq t_{l}^{\sigma ,v}<{rt}_{p_{k+1}^{\sigma }}\;\&\;{uf}_{t_{l}^{\sigma ,v}}\cdot {ur}_{t_{l}^{\sigma ,v}}>\sum_{\phi^{\sigma }}{ub}_{t_{l}^{\sigma ,v}}^{\phi^{\sigma }}\\ 0 & others \end{array}\right.,\;1\leq j\leq e^{\sigma ,v},\;1\leq l\leq e^{\sigma ,v},\;1\leq k<h^{\sigma }$$

(50) $$\beta_{t_{j}^{\sigma ,v}}=\left\{\begin{array}{cc} 1 & \;{if\;rt}_{p_{k}^{\sigma }}\leq t_{l}^{\sigma ,v}<{rt}_{p_{k+1}^{\sigma }}\;\&\;{df}_{t_{l}^{\sigma ,v}}\cdot {dr}_{t_{l}^{\sigma ,v}}>\sum_{\phi^{\sigma }}{db}_{t_{l}^{\sigma ,v}}^{\phi^{\sigma }}\\ 0 & others \end{array}\right.,\;1\leq j\leq e^{\sigma ,v},\;1\leq l\leq e^{\sigma ,v},\;1\leq k<h^{\sigma }$$

(51) $$\sum_{s=1}^{S^{\sigma ,v}}\sum_{\sigma^{r_{s,1}^{\sigma ,v}}}{ub}_{t_{l}^{\sigma ,v}}^{\varphi^{r_{s,1}^{\sigma ,v}}}\geq {uf}_{t_{l}^{\sigma ,v}}\cdot {ur}_{t_{l}^{\sigma ,v}}>\sum_{\phi^{\sigma }}{ub}_{t_{l}^{\sigma ,v}}^{\phi^{\sigma }},\;1\leq l\leq e^{\sigma ,v}\;{if\;rt}_{p_{k}^{\sigma }}\leq t_{l}^{\sigma ,v}<{rt}_{p_{k+1}^{\sigma }}\;\&\gamma^{\sigma ,v}=1,$$

(52) $$\sum_{s=1}^{S^{\sigma ,v}}\sum_{\varphi^{r_{s,1}^{\sigma ,v}}}{db}_{t_{l}^{\sigma ,v}}^{\sigma^{r_{s,1}^{\sigma ,v}}}\geq {df}_{t_{l}^{\sigma ,v}}\cdot {dr}_{t_{l}^{\sigma ,v}}>\sum_{\phi^{\sigma }}{db}_{t_{l}^{\sigma ,v}}^{\phi^{\sigma }},\;1\leq l\leq e^{\sigma ,v}\;{if\;rt}_{p_{k}^{\sigma }}\leq t_{l}^{\sigma ,v}<{rt}_{p_{k+1}^{\sigma }},$$

(53) $${urb}_{t_{l}^{\sigma ,v}}^{r_{s,i}^{\sigma ,v},r_{s,i+1}^{\sigma ,v}}\geq {uvb}_{t_{l}^{\sigma ,v}}^{r_{s,i}^{\sigma ,v},r_{s,i+1}^{\sigma ,v}},\;1\leq l\leq e^{\sigma ,v},1\leq s\leq S_{k}^{\sigma ,v},1\leq i<e^{\sigma ,v}\;if\;\gamma^{\sigma ,v}=1$$

(54) $${drb}_{t_{l}^{\sigma ,v}}^{r_{s,i}^{\sigma ,v},r_{s,i+1}^{\sigma ,v}}\geq {dvb}_{t_{l}^{\sigma ,v}}^{r_{s,i}^{\sigma ,v},r_{s,i+1}^{\sigma ,v}},\;1\leq l\leq e^{\sigma ,v},1\leq i<e^{\sigma ,v},1\leq s\leq S_{k}^{\sigma ,v},$$

(55) $$t_{l+1}^{\sigma ,v}=t_{l}^{\sigma ,v}+∆,\;1\leq l\leq e^{\sigma ,v}$$

where $R_{s}^{\sigma ,v}$ represents the *s*-th fleet capable of forwarding bandwidth for application v, $S^{\sigma ,v}$ is the number of fleets supporting v, and the length of fleet *s* is denoted by $\eta_{s}^{\sigma ,v}$. $r_{s,1}^{\sigma ,v}$ and $r_{s,\eta_{s}^{\sigma ,v}}^{\sigma ,v}$ represent the EV indexes at the beginning and end of the fleet *s*, respectively. $\varphi^{r_{s,1}^{\sigma ,v}}$ and $\varphi^{\sigma }$ represent the index value of the CF mMIMO cluster member or base station located at the origin of the fleet, respectively. $p_{h^{\sigma }}^{\sigma }$ is the destination of EV σ, and σ cannot support the required bandwidth for v when passing through the *k*-th segment between $p_{k}^{\sigma }$ and $p_{k+1}^{\sigma }$. The symbols $t_{1}^{\sigma ,v}$ and $t_{e^{\sigma ,v}}^{\sigma ,v}$, respectively, represent the times when v starts and ends. When the binary flag $\gamma^{\sigma ,v}$ is set to 1, it signifies that *v* is bidirectional, while the binary flags $\alpha_{t_{j}^{\sigma ,v}}$ and $\beta_{t_{j}^{\sigma ,v}}$ represent the segment where the bandwidth for uploading and downloading v are inadequate at time $t_{j}^{\sigma ,v}$, respectively. ${urb}_{t_{l}^{\sigma ,v}}^{r_{s,i}^{\sigma ,v},r_{s,i+1}^{\sigma ,v}}$ and ${drb}_{t_{l}^{\sigma ,v}}^{r_{s,i}^{\sigma ,v},r_{s,i+1}^{\sigma ,v}}$ stand for the remaining bandwidth available for upload and download from EV $r_{s,i}^{\sigma ,v}$ to EV $r_{s,i+1}^{\sigma ,v}$ at time $t_{l}^{\sigma ,v}$, respectively, while ${uvb}_{t_{l}^{\sigma ,v}}^{r_{s,i}^{\sigma ,v},r_{s,i+1}^{\sigma ,v}}$ and ${dvb}_{t_{l}^{\sigma ,v}}^{r_{s,i}^{\sigma ,v},r_{s,i+1}^{\sigma ,v}}$, respectively, represent the total upload and download bandwidth from EV $r_{s,i}^{\sigma ,v}$ to EV $r_{s,i+1}^{\sigma ,v}$.

Step 2: If the preceding step fails to form any fleet capable of supporting the required bandwidth for v, notify the RSU that initiated this module and terminate the execution of this module.

Step 3: The required bandwidth for v is transmitted from base stations or CF mMIMO clusters to the fleet using Li-Fi, enabling V2V communication.

Step 4: Whether EV σ passing through a segment still cannot meet the bandwidth requirements for v can be checked as follows:

(56) $${dub}_{t_{l}^{\sigma ,v}}=\left\{\begin{array}{cc} {uf}_{t_{l}^{\sigma ,v}}\cdot {ur}_{t_{l}^{\sigma ,v}}-\sum_{s=1}^{S^{\sigma ,v}}\sum_{\varphi^{r_{s,1}^{\sigma ,v}}}{ub}_{t_{l}^{\sigma ,v}}^{\sigma^{r_{s,1}^{\sigma ,v}}} & \;if\;\alpha_{t_{j}^{\sigma ,v}}=1\;\&\sum_{s=1}^{S^{\sigma ,v}}\sum_{\varphi^{r_{s,1}^{\sigma ,v}}}{ub}_{t_{l}^{\sigma ,v}}^{\sigma^{r_{s,1}^{\sigma ,v}}}-{uf}_{t_{l}^{\sigma ,v}}\cdot {ur}_{t_{l}^{\sigma ,v}}<0\\ 0 & others \end{array}\right.,$$

(57) $${ddb}_{t_{l}^{\sigma ,v}}=\left\{\begin{array}{cc} {df}_{t_{l}^{\sigma ,v}}\cdot {ur}_{t_{l}^{\sigma ,v}}-\sum_{s=1}^{S^{\sigma ,v}}\sum_{\varphi^{r_{s,1}^{\sigma ,v}}}{db}_{t_{l}^{\sigma ,v}}^{\varphi^{r_{s,1}^{\sigma ,v}}} & \;if\;\alpha_{t_{j}^{\sigma ,v}}=1\;\&\sum_{s=1}^{S^{\sigma ,v}}\sum_{\varphi^{r_{s,1}^{\sigma ,v}}}{db}_{t_{l}^{\sigma ,v}}^{\varphi^{r_{s,1}^{\sigma ,v}}}-{df}_{t_{l}^{\sigma ,v}}\cdot {dr}_{t_{l}^{\sigma ,v}}<0\\ 0 & others \end{array}\right.,$$

where $t_{j}^{\sigma ,v}$ represents the time period during which the bandwidth is insufficient for v, and ${dub}_{t_{l}^{\sigma ,v}}$ and ${ddb}_{t_{l}^{\sigma ,v}}$ denote the bandwidth deficit for upload and download during $t_{j}^{\sigma ,v}$, respectively.

Step 5: The bandwidth forwarding result is communicated back to the RSU that initiated this module.

## 3. Analysis and Discussion of Simulation Results

A simulation was conducted on a personal computer equipped with an Intel Core i7 2.9 GHz CPU and 64 GB RAM to evaluate the proposed algorithm. Because communication technologies like 6G and CF mMIMO are still in their early development stages, actual data are not yet available. Consequently, this study relies on numerical validation to assess the effectiveness of the proposed algorithm.

This work references the vehicle volume statistics for New York City [35]. The starting and ending points for EVs are randomly generated. The total number of vehicles on the road at any given time is aligned with the traffic density data from [35]. Since a dedicated EV database is not available, this study treats the vehicles referenced in [35] as EVs.

The multimedia applications are categorized into three types: non-real-time, real-time, and emergency applications. Emergency applications, including 360° video calls, use HEVC with reduced encoding complexity. Real-time applications, such as 2D video calls, 2D live video, and 360° live video, also use HEVC with lower encoding complexity. Non-real-time applications, including 2D VoD and 360° VoD, employ VVC at half the bitrate of HEVC. The usage proportions and frequencies of different video types in this study are estimated from data provided by the websites referenced in [36,37,38,39,40,41,42].

The parameters used in the proposed algorithm are detailed below. The time slot interval was set to 10 s, and each video segment had a duration of 1000 milliseconds. It was assumed that each EV had storage capacity well beyond the size of the preloaded video segments. Moreover, the number of passengers per EV varied, with possible values ranging from 1 to 5.

Table 1, Table 2 and Table 3 present the anticipated bandwidth demands for various types of 2D videos [43] and 360° videos [44]. The projected bandwidth requirements for non-real-time applications are detailed in Table 1, with VVC using half the bandwidth of HEVC for 2D and 360° VoD. Table 2 and Table 3 present the projected bandwidth requirements for real-time applications and emergency applications using HEVC, respectively, where 2D video call and 360° video call involve bidirectional transmission.

The simulation environment utilized three types of cellular base stations, sub-6GHz [45], mmWave [45,46], and THz [45,47], along with WiFi 7 [20,46] access points in a non-cellular architecture, to provide bandwidth for various applications used by EV users. Furthermore, LiFi [48] technology was implemented for V2V communication among EVs. Table 4 details these wireless communication technologies’ maximum transmission distance and bandwidth.

**Figure 6:**
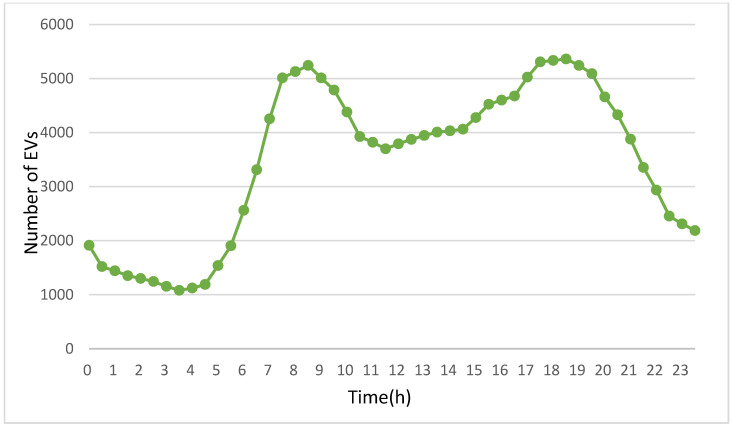
Volume of EVs throughout a day.

Figure 6 depicts the daily fluctuations in the number of EVs on the road, with vehicle counts based on traffic density data from [35]. The vertical axis shows the number of EVs, while the horizontal axis indicates the time in hourly units. The data are presented in 30 min intervals, with hourly markers on the horizontal axis for clarity. As revealed in Figure 6, a sharp increase in the number of EVs is observed after 5 a.m. as people begin their morning commutes, continuing to surge until peaking at 8:30 a.m., coinciding with the typical start of the workday for many commuters.

Following the 8:30 a.m. peak, the number of vehicles decreases but then stabilizes, maintaining a consistent level of around 4000 vehicles between 10:30 a.m. and 3 p.m. This steady state suggests a period of relative equilibrium in vehicle usage, likely due to people being at work and making fewer trips. In the late afternoon, starting around 3 p.m., there is another rise in the number of EVs. This second increase aligns with the end of the workday, as people begin their commutes back home or to other evening activities. The vehicle count reaches another peak at 6 p.m., reflecting the high volume of evening travel. After this evening peak, the number of vehicles on the road begins to decline again. This downward trend continues through the night, reaching its lowest point during the early morning hours. This pattern suggests that most EVs are idle from late night to early morning, likely because people are at home instead of traveling or the vehicles are being charged during these hours.

**Figure 7:**
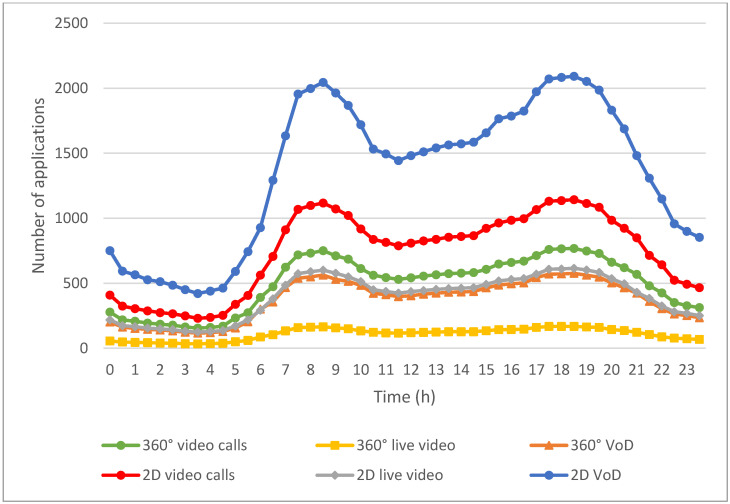
Counts of multimedia applications initiated by passengers in EVs throughout a day.

Figure 7 illustrates the usage of multimedia applications by EV users throughout the day. The usage numbers for each application are estimated from data provided by the websites referenced in [36,37,38,39,40,41,42] and adjusted according to the fluctuations in the number of EVs during different time periods. These applications fall into six categories: emergency applications, such as 360° video calls; real-time applications, including 2D video calls, 2D live video, and 360° live video; and non-real-time applications, such as 2D VoD and 360° VoD. The usage patterns of these multimedia applications are closely linked to the fluctuations in the number of EVs depicted in Figure 6, indicating a strong correlation between vehicle activity and multimedia consumption.

As illustrated in Figure 7, the low number of EVs correlates with lower multimedia application usage in the early morning hours. This is likely due to fewer people being on the road and therefore less demand for entertainment or communication via multimedia applications. As the number of vehicles rises from 5 a.m. onwards, corresponding to the start of the morning commute, the usage of all applications increases. This increase continues until it peaks during the morning rush hour at 8:30 a.m. Users likely engage with multimedia applications to kill time, stay informed, or be entertained while the EV is in motion.

After the morning peak, multimedia application usage tends to decrease as the number of EVs stabilizes. However, the number of EVs starts to rise again at around 3 p.m., and there is a corresponding increase in multimedia application usage. This trend continues until it reaches another peak during the evening rush hour at 6 PM, similar to the morning peak. During these times, users frequently engage with multimedia applications, whether during their commutes home or while participating in evening activities.

Notably, VoD applications are more frequently used throughout the day compared to live video and emergency applications. The flexibility of VoD allows users to pause, fast-forward, and replay content at their convenience, which is particularly appealing for commuters with unpredictable schedules. Additionally, the wider variety of content available on VoD platforms attracts a larger audience.

In contrast, live video applications are used less frequently because they are tied to specific broadcast times, making them less convenient for users with varying schedules. Furthermore, 360° videos are less commonly used compared to 2D videos due to their higher production complexity and greater network requirements. The immersive nature of 360° videos, while appealing, demands more bandwidth and better connectivity, which may not always be available to EV users on the go.

**Figure 8:**
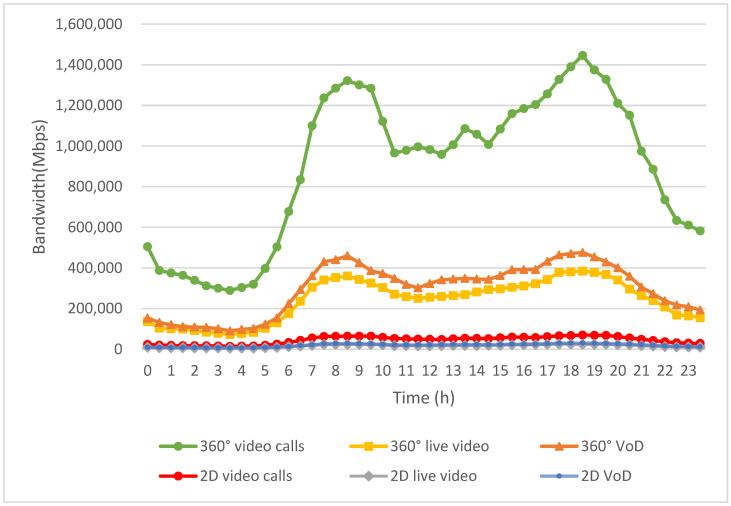
Daily bandwidth demands of multimedia applications.

Figure 8 illustrates the bandwidth requirements for six different multimedia applications: 2D VoD, 360° VoD, 2D live video, 360° live video, 2D video calls, and 360° video calls. These requirements are determined based on the usage numbers of multimedia applications during different time periods. The video resolution and frame rate for each application type are randomly selected from the corresponding video categories in Table 1, Table 2 and Table 3. As illustrated by the red, gray, and blue curves in Figure 8, the bandwidth demand for 2D video is relatively low, as outlined in Table 1 and Table 2, even though 2D video is more frequently used. In other words, the bandwidth needs for 2D video calls, 2D live video, and 2D VoD are noticeably lower than those for the three 360° video applications.

As shown by the green, tangerine, and yellow curves in Figure 8, 360° videos demand significant bandwidth to deliver immersive 3D content, providing users with a more engaging experience. As a result, the bandwidth requirements for 360° VoD, 360° live video, and 360° video calls are higher compared to those for 2D videos. This increased demand is due to the higher resolution and larger data volumes needed to create the 360° field of view, which enhances user immersion but also significantly increases the amount of data that must be transmitted.

Moreover, non-real-time applications, like 2D VoD and 360° VoD, use highly compressed VVC to minimize bandwidth usage. This advanced compression technology allows for high-quality video playback without requiring excessive data transmission. Consequently, the bandwidth demand for VoD, despite its higher viewing rates, is not significantly greater than that of other applications. This efficiency in data compression means that users can enjoy high-quality video content with less strain on network resources.

Conversely, real-time applications, such as video calls, necessitate the simultaneous uploading and downloading of video streams, which require more bandwidth. This bidirectional data flow leads to relatively higher bandwidth demands compared to VoD applications. Specifically, 2D video calls, while less bandwidth-intensive than their 360° counterparts, still require more bandwidth than VoD due to the need for real-time interaction and low latency to maintain a seamless communication experience.

The disparity in bandwidth requirements between 2D and 360° applications is further accentuated in live video scenarios, as shown in Figure 8; 360° live video not only demands high bandwidth for transmitting large amounts of data in real-time but also requires robust network infrastructure to handle the simultaneous streaming to multiple users. This makes 360° live video one of the most bandwidth-intensive applications, necessitating advanced network solutions to support widespread use.

**Figure 9:**
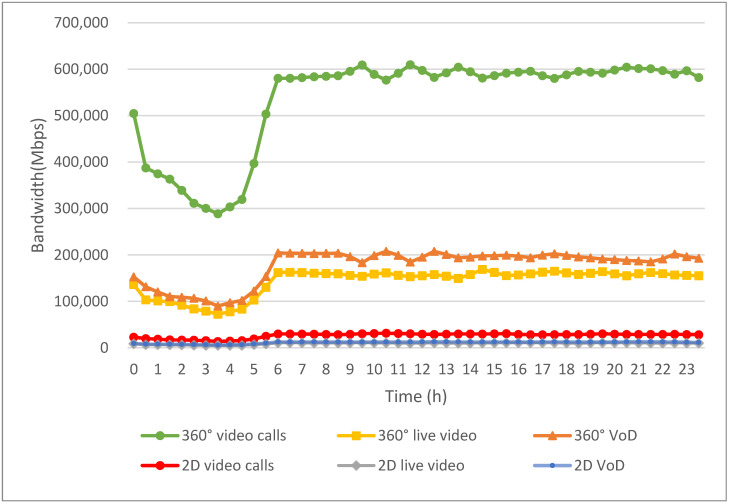
Bandwidth assignments for multimedia applications before implementing the proposed work.

Figure 9 illustrates the bandwidth allocated to each multimedia application before implementing the algorithm proposed in this study. In the early morning hours, when user bandwidth demand is low, the bandwidth requirements for all applications are adequately met. However, as the day progresses and the number of EVs increases, the use of multimedia applications grows correspondingly, resulting in a substantial rise in bandwidth demand. This escalation leads to periods during the daytime when users experience insufficient bandwidth availability.

As depicted in Figure 9, due to the lower number of active EVs and the resulting minimal usage of multimedia applications during the early morning hours, the overall demand for bandwidth remains manageable. This period typically experiences surplus bandwidth, ensuring that users can access their desired content without interruption.

However, starting from 5 a.m., the increase in active EVs is accompanied by a rise in the usage of multimedia applications, such as live video, VoD, and video calls. This surge in demand continues throughout the morning rush hour, peaking at around 8:30 a.m., when the need for bandwidth reaches its highest point due to widespread commuter activity and multimedia consumption.

Notably, starting from around 6 a.m., the bandwidth-allocation curve tends to flatten out. During this period, there is a sustained and significant demand for multimedia applications. However, the allocated bandwidth for EV passengers often reaches its maximum limit due to the confined bandwidth supply in vehicular networks.

Despite the overall increase in bandwidth demand throughout the day, Figure 9 illustrates that there are periods when the allocated bandwidth may not sufficiently meet the needs of all users. This discrepancy can lead to degraded service quality, such as buffering during video playback or dropped connections during video calls, impacting user experience.

**Figure 10:**
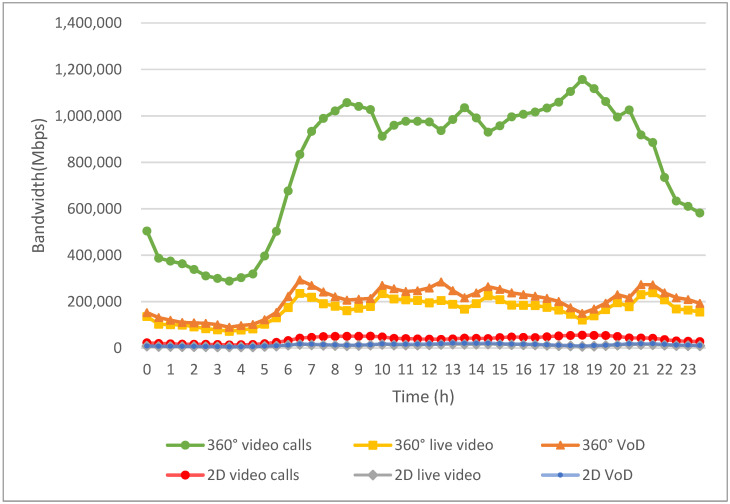
Bandwidth assignments for multimedia applications after implementing the proposed work.

Figure 10 visualizes the improved approach to bandwidth allocation implemented through our algorithm, demonstrating significant improvements across various multimedia applications. The improvements are attributed to several key modules of the proposed work that aim to optimize bandwidth utilization and enhance user experience in diverse usage scenarios.

A standout feature of the proposed work is the “Emergency/Real-time Application Bandwidth Assignment” module, which prioritizes bandwidth allocation for critical applications, such as 360° video calls from CF mMIMO access points. Additionally, when emergency applications cannot obtain sufficient bandwidth, the module reallocates bandwidth that was originally designed for non-real-time applications to emergency applications. This module ensures that even during peak demand periods, resources are allocated efficiently, significantly enhancing the availability of bandwidth for emergency application needs.

Additionally, the algorithm incorporates the “V2V Bandwidth Forwarding Module”, leveraging V2V communication to dynamically redistribute bandwidth. By utilizing connectivity between nearby vehicles, this module optimizes the allocation of bandwidth from base stations and access points across different areas. This approach maximizes the fulfillment of bandwidth demands for all multimedia applications, ensuring consistent performance and reliability throughout EV environments.

Overall, Figure 10 underscores how the implemented algorithm revolutionizes bandwidth management in EV environments. By prioritizing critical applications, optimizing resource allocation through V2V communication, the algorithm significantly enhances bandwidth availability and improves the overall multimedia experience for users. These advancements are essential for meeting the increasing demands of multimedia applications in dynamic and densely populated EV settings, ensuring reliable and efficient multimedia usage across various scenarios.

**Figure 11:**
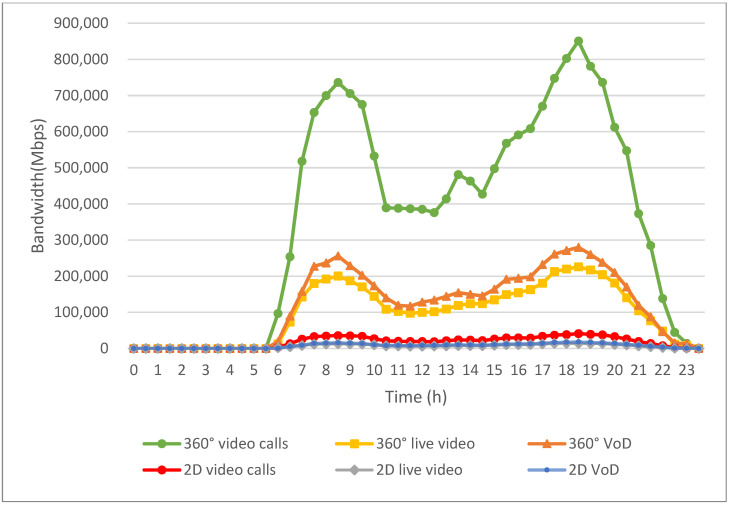
Difference between allocated bandwidth and expected bandwidth needs before implementing the proposed algorithm.

Figure 11 illustrates the gap between the actual bandwidth allocated to EV users’ applications and their expected bandwidth requirements before the implementation of the proposed work. As observed in Figure 11, the bandwidth needs for all application types are met from 11:30 p.m. to 5:30 a.m. However, as user bandwidth demand increases, the disparity between the available bandwidth and the expected requirements widens.

Prior to implementing the algorithm, bandwidth allocation followed a first-come, first-served approach, which did not account for bandwidth-allocation priorities. Consequently, the largest gap is seen in the emergency application, 360° video calls, which has the highest bandwidth demand. In contrast, the gap for 2D video, which requires less bandwidth, is less significant.

**Figure 12:**
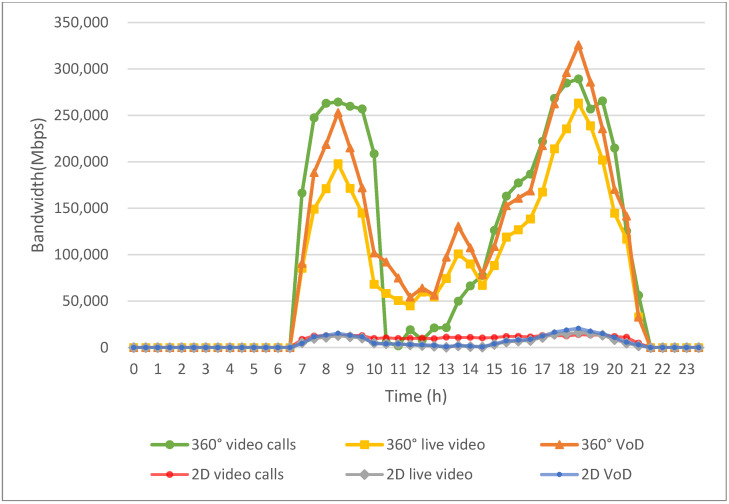
Difference between allocated bandwidth and expected bandwidth needs after implementing the proposed algorithm.

Figure 12 highlights the differences between the allocated bandwidth and the expected bandwidth requirements for EV users’ applications after the implementation of the proposed work. With our proposed algorithm in place, there is a noticeable improvement in the bandwidth assigned to EV users’ applications. From 9:30 p.m. to 6:30 a.m., applications’ bandwidth needs are consistently satisfied. Even during peak demand periods, the difference between the assigned bandwidth and applications’ needs is substantially reduced, attributed to the V2V bandwidth forwarding strategy that enhances overall bandwidth utilization.

Additionally, Figure 12 illustrates a significant reduction in the bandwidth gap for emergency applications, such as 360° video calls. This improvement is achieved by prioritizing bandwidth usage for emergency applications and reallocating bandwidth from other types of applications to emergency ones. Other applications also benefit from V2V communication, which redistributes bandwidth from less congested base stations or access points at less congested road segments, thereby reducing the gap between assigned bandwidth and their requirements.

**Figure 13:**
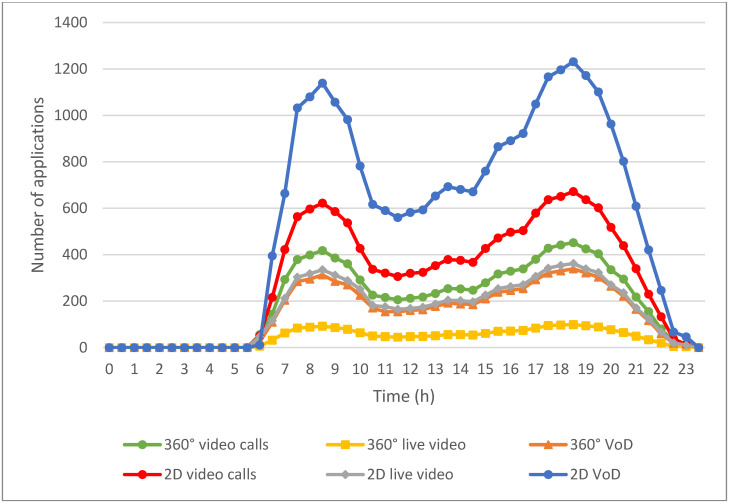
Number of playback interruptions for each type of application before implementing the proposed algorithm.

Figure 13 illustrates the number of playback interruptions for each type of application caused by insufficient bandwidth before implementing the algorithm. During the period from 11:30 p.m. to 5:30 a.m., when bandwidth requirements are generally met, users receive adequate bandwidth to ensure the smooth playback of their applications. This period sees minimal interruptions, indicating that the existing bandwidth allocation is sufficient for the user demand during these hours.

However, as the number of EV users increases during the morning commute, bandwidth resource contention becomes a significant issue. The growing demand for bandwidth leads to more EV users’ applications being unable to acquire sufficient bandwidth, resulting in a marked increase in playback interruptions across various applications. This deteriorated quality of EV users’ experiences underscores the need for a more efficient bandwidth-allocation strategy.

**Figure 14:**
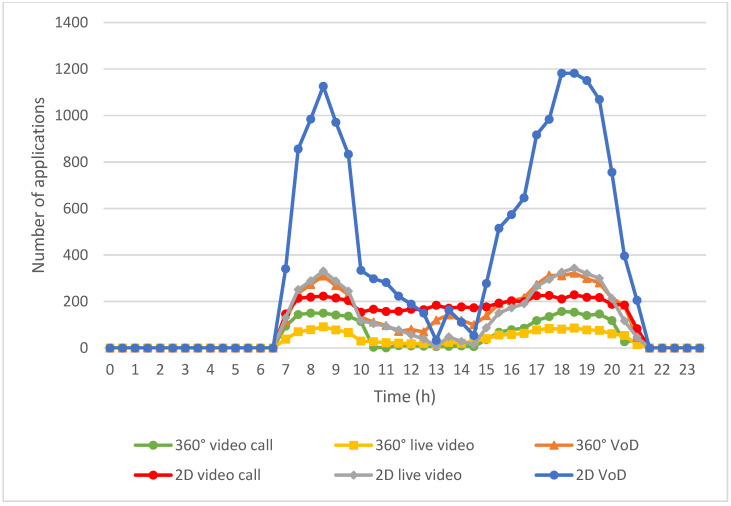
Number of playback interruptions for each type of application after implementing the algorithm.

Figure 14 contrasts the number of playback interruptions for each type of application after implementing the proposed algorithm. A significant reduction in interruptions is observed across all time periods for most of EV users’ applications. Specifically, during the morning and evening peak demand periods, five of the six types of applications see a substantial decline in the number of playback interruptions, while only 2D VoD shows a slight decrease in playback interruptions.

Notably, the interruptions for emergency applications, such as 360° video calls, approach zero between 9:30 p.m. and 6:30 a.m. and between 10:30 a.m. and 2:30 p.m. This represents the lowest interruption rate among all types of multimedia applications. This improvement is mainly achieved by prioritizing bandwidth allocation for different applications, effectively minimizing interruptions for emergency and real-time applications.

In addition, the “Multimedia Application Bandwidth Adjustment” module adjusts parameters, such as the frame rate and resolution, based on the type of multimedia application to meet the specific needs of EV users’ applications. Consequently, even during peak bandwidth demand periods, when EV users’ applications experience a bandwidth deficit, this module reduces the frame rate and resolution of the applications to decrease the bandwidth required. This adjustment effectively prevents interruptions or buffering during multimedia usage, thereby improving the overall user experience.

**Figure 15:**
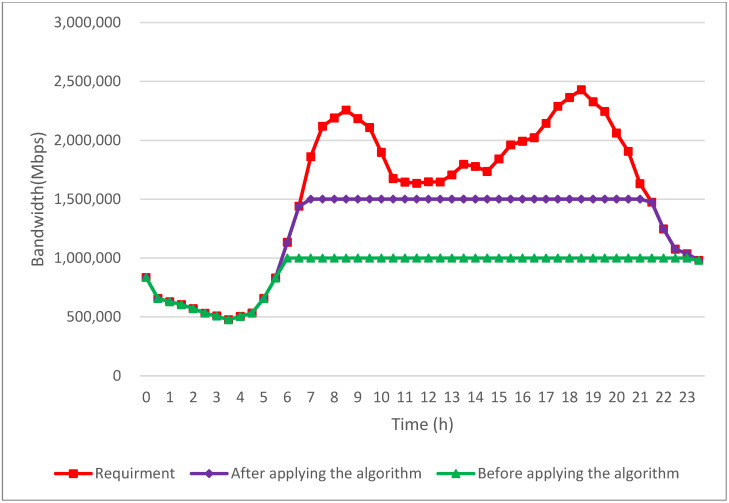
Contrast of bandwidth requirements and assigned bandwidth before and after algorithm implementation.

Figure 15 presents a comparison of the overall bandwidth requirements for all multimedia applications during a day before and after the implementation of the proposed work. During the early hours, from midnight to 5:30 a.m., bandwidth demand remains relatively low across all multimedia applications. Consequently, existing bandwidth resources generally meet these demands adequately without requiring intervention from the proposed algorithm.

However, as time progresses, typically starting around the morning hours, the bandwidth demand of applications begins to increase. This increase in demand can escalate rapidly, especially during peak periods, such as morning and evening rush hours. Often, this surge surpasses the maximum capacity of congested CF mMIMO wireless access points and base stations, resulting in insufficient bandwidth for many EV passengers’ applications. This creates a notable disparity between the demand for bandwidth and its availability, particularly in densely populated or high-traffic areas.

Before the implementation of our proposed algorithm, bandwidth was allocated using a first-come, first-served approach. This method did not prioritize critical applications and lacked a V2V bandwidth scheduling strategy, often leading to insufficient bandwidth for emergency applications and the inefficient use of available resources. On the contrary, the algorithm introduced in this study effectively addresses these bandwidth limitations within congested V2X networks. A critical mechanism is the V2V bandwidth scheduling, facilitated by LiFi technology, which dynamically redistributes bandwidth. This approach optimizes resource use by reallocating bandwidth from less congested w CF mMIMO wireless access points and base stations to vehicles on congested roads via V2V communication. By leveraging V2V communication, the algorithm efficiently schedules bandwidth, ensuring that EV users on busy routes have access to enhanced resources.

Facilitated by the proposed algorithm, there is a substantial increase in available bandwidth for EV users’ applications, particularly noticeable from 6 a.m. to 23 p.m. as shown in Figure 15. A 47% increase is achieved in assigned bandwidth, effectively mitigating network resource contention issues and improving the overall user experience with reliable multimedia application performance. Figure 15 also demonstrates how the proposed algorithm revolutionizes bandwidth management in congested V2X networks. By optimizing resource allocation through advanced V2V communication and LiFi technology and efficiently managing CF mMIMO access points and base station allocated bandwidth, the algorithm significantly enhances bandwidth-utilization efficiency. This proactive approach effectively addresses bandwidth limitations, resulting in an enriched multimedia experience for EV users across various usage scenarios and timeframes.

## 4. Conclusions

Although numerous studies have proposed algorithms for multimedia transmission in V2X networks, the potential of emerging wireless communication technologies remains largely unexplored. This study aims to integrate various cutting-edge technologies, including THz, mmWave, sub-6GHz cellular base stations, and CF mMIMO Wi-Fi 7 access points. The objective is to deliver ultra-high transmission rates, seamless connectivity, and minimal interference for EV users in V2X networks. Additionally, the study investigates the use of Li-Fi technology for vehicular applications, leveraging it to enhance V2V communication and meet the bandwidth requirements of other vehicles.

This study validates the proposed mechanisms through a detailed simulation analysis, showing an average bandwidth increase of 47% for EV users’ applications during peak times. The proposed mechanisms include prioritizing bandwidth for emergency and real-time applications, implementing a pre-downloading strategy for non-real-time applications, and using V2V communication for bandwidth relay from less congested access points or base stations. This approach ensures that EV users’ applications have adequate bandwidth during high-demand periods, facilitating smooth multimedia playback.

In summary, compared to the existing literature, this study introduces a range of emerging wireless technologies into V2X networks, significantly boosting user bandwidth through V2V communication and addressing the prioritization of various application types. The proposed algorithm effectively manages bandwidth contention among EV passengers and ensures uninterrupted multimedia application playback during peak demand. By meeting the needs of most EV passengers, the algorithm enhances overall service satisfaction and positions itself as a leader in bandwidth management within the evolving field of EV connectivity.

Due to the absence of actual data for testing, this study is limited to numerical validation. Once actual data become accessible in the future, further validation will be conducted to verify the feasibility of the proposed algorithm. Moreover, as EV adoption becomes more prevalent, incorporating real-world data on EV charging times into the simulations will be considered to enhance their accuracy and relevance to actual conditions.

## Figures and Tables

**Table 1:** Anticipated bandwidth demands for non-real-time applications using VVC.

Video Type	Video Resolution/Frame Rate	Anticipated Download Bandwidth
2D VoD	720 p 60 fps	4 Mbps
	1080 p 60 fps	5 Mbps
	1440 p 60 fps	15 Mbps
	2160 p 60 fps	20 Mbps
360° VoD	4 K 30 fps	12.5 Mbps
	8 K 30 fps	50 Mbps
	12 K 60 fps	200 Mbps
	24 K 120 fps	1.175 Gbps

**Table 2:** Anticipated bandwidth requirements for real-time applications using HEVC.

Video Type	Video Resolution/Frame Rate	Anticipated Download Bandwidth	Anticipated Upload Bandwidth
2D live video	720 p 60 fps	8 Mbps	✕
	1080 p 60 fps	10 Mbps	✕
	1440 p 60 fps	30 Mbps	✕
	2160 p 60 fps	40 Mbps	✕
360° live video	4 K 30 fps	25 Mbps	✕
	8 K 30 fps	100 Mbps	✕
	12 K 60 fps	400 Mbps	✕
	24 K 120 fps	2.35 Gbps	✕
2D video call	720 p 60 fps	8 Mbps	8 Mbps
	1080 p 60 fps	10 Mbps	10 Mbps
	1440 p 60 fps	30 Mbps	30 Mbps
	2160 p 60 fps	40 Mbps	40 Mbps

**Table 3:** Anticipated bandwidth requirements for emergency applications using HEVC.

Video type	Video Resolution/Frame Rate	Anticipated Download Bandwidth	Anticipated Upload Bandwidth
360° video call	4 K 30 fps	25 Mbps	25 Mbps
	8 K 30 fps	100 Mbps	100 Mbps
	12 K 60 fps	400 Mbps	400 Mbps
	24 K 120 fps	2.35 Gbps	2.35 Gbps

**Table 4:** Maximum communication distance and bandwidth for these wireless communication technologies.

Type of Wireless Communication Technology	Maximum Communication Distance	Maximum Bandwidth
sub-6GHz	620 m	6.5 Gbps
mmWave	200 m	10 Gbps
THz	20 m	100 Gbps
WiFi 7	300 m	46.1 Gbps
Li-Fi	10 m	640 Gbps

## Data Availability

Simulation data are available to collaborating researchers in coordination with the corresponding author.
